# Maturation of hiPSC-derived cardiomyocytes promotes adult alternative splicing of SCN5A and reveals changes in sodium current associated with cardiac arrhythmia

**DOI:** 10.1093/cvr/cvac059

**Published:** 2022-04-08

**Authors:** Giulia Campostrini, Georgios Kosmidis, Dorien Ward-van Oostwaard, Richard Paul Davis, Loukia Yiangou, Daniele Ottaviani, Christiaan Cornelis Veerman, Hailiang Mei, Valeria Viktorovna Orlova, Arthur Arnold Maria Wilde, Connie Rose Bezzina, Arie Otto Verkerk, Christine Lindsay Mummery, Milena Bellin

**Affiliations:** Department of Anatomy and Embryology, Leiden University Medical Center (LUMC), 2333 ZA Leiden, The Netherlands; Department of Anatomy and Embryology, Leiden University Medical Center (LUMC), 2333 ZA Leiden, The Netherlands; Department of Anatomy and Embryology, Leiden University Medical Center (LUMC), 2333 ZA Leiden, The Netherlands; Department of Anatomy and Embryology, Leiden University Medical Center (LUMC), 2333 ZA Leiden, The Netherlands; Department of Anatomy and Embryology, Leiden University Medical Center (LUMC), 2333 ZA Leiden, The Netherlands; Department of Biology, University of Padua, 35121 Padua, Italy; Veneto Institute of Molecular Medicine, 35129 Padua, Italy; Department of Clinical and Experimental Cardiology, Heart Centre, Amsterdam University Medical Centre, location AMC, University of Amsterdam, Meibergdreef 9, 1105 AZ, Amsterdam, The Netherlands; Sequencing Analysis Support Core, Leiden University Medical Center, 2333 Leiden, The Netherlands; Department of Anatomy and Embryology, Leiden University Medical Center (LUMC), 2333 ZA Leiden, The Netherlands; Department of Clinical and Experimental Cardiology, Heart Centre, Amsterdam University Medical Centre, location AMC, University of Amsterdam, Meibergdreef 9, 1105 AZ, Amsterdam, The Netherlands; Department of Clinical and Experimental Cardiology, Heart Centre, Amsterdam University Medical Centre, location AMC, University of Amsterdam, Meibergdreef 9, 1105 AZ, Amsterdam, The Netherlands; Department of Clinical and Experimental Cardiology, Heart Centre, Amsterdam University Medical Centre, location AMC, University of Amsterdam, Meibergdreef 9, 1105 AZ, Amsterdam, The Netherlands; Department of Medical Biology, Amsterdam Cardiovascular Sciences, Amsterdam UMC, University of Amsterdam, Meibergdreef 9, 1105 AZ, Amsterdam, The Netherlands; Department of Anatomy and Embryology, Leiden University Medical Center (LUMC), 2333 ZA Leiden, The Netherlands; Department of Applied Stem Cell Technologies, University of Twente, 7500 AE, Enschede, The Netherlands; Department of Anatomy and Embryology, Leiden University Medical Center (LUMC), 2333 ZA Leiden, The Netherlands; Department of Biology, University of Padua, 35121 Padua, Italy; Veneto Institute of Molecular Medicine, 35129 Padua, Italy

**Keywords:** human-induced pluripotent stem cell-derived cardiomyocytes, cardiac sodium channel, SCN5A, cardiac arrhythmias, cardiac microtissue

## Abstract

**Aims:**

Human-induced pluripotent stem cell-cardiomyocytes (hiPSC-CMs) are widely used to study arrhythmia-associated mutations in ion channels. Among these, the cardiac sodium channel *SCN5A* undergoes foetal-to-adult isoform switching around birth. Conventional hiPSC-CM cultures, which are phenotypically foetal, have thus far been unable to capture mutations in adult gene isoforms. Here, we investigated whether tri-cellular cross-talk in a three-dimensional (3D) cardiac microtissue (MT) promoted post-natal *SCN5A* maturation in hiPSC-CMs.

**Methods and results:**

We derived patient hiPSC-CMs carrying compound mutations in the adult *SCN5A* exon 6B and exon 4. Electrophysiological properties of patient hiPSC-CMs in monolayer were not altered by the exon 6B mutation compared with isogenic controls since it is not expressed; further, CRISPR/Cas9-mediated excision of the foetal exon 6A did not promote adult *SCN5A* expression. However, when hiPSC-CMs were matured in 3D cardiac MTs, *SCN5A* underwent isoform switch and the functional consequences of the mutation located in exon 6B were revealed. Up-regulation of the splicing factor muscleblind-like protein 1 (*MBNL1*) drove *SCN5A* post-natal maturation in microtissues since its overexpression in hiPSC-CMs was sufficient to promote exon 6B inclusion, whilst knocking-out *MBNL1* failed to foster isoform switch.

**Conclusions:**

Our study shows that (i) the tri-cellular cardiac microtissues promote post-natal *SCN5A* isoform switch in hiPSC-CMs, (ii) adult splicing of *SCN5A* is driven by MBNL1 in these tissues, and (iii) this model can be used for examining post-natal cardiac arrhythmias due to mutations in the exon 6B.

**Translational perspective:**

The cardiac sodium channel is essential for conducting the electrical impulse in the heart. Postnatal alternative splicing regulation causes mutual exclusive inclusion of fetal or adult exons of the corresponding gene, SCN5A. Typically, immature hiPSCCMs fall short in studying the effect of mutations located in the adult exon. We describe here that an innovative tri-cellular three-dimensional cardiac microtissue culture promotes hiPSC-CMs maturation through upregulation of MBNL1, thus revealing the effect of a pathogenic genetic variant located in the SCN5A adult exon. These results help advancing the use of hiPSC-CMs in studying adult heart disease and for developing personalized medicine applications.

## Graphical Abstract

**Graphical Abstract:**
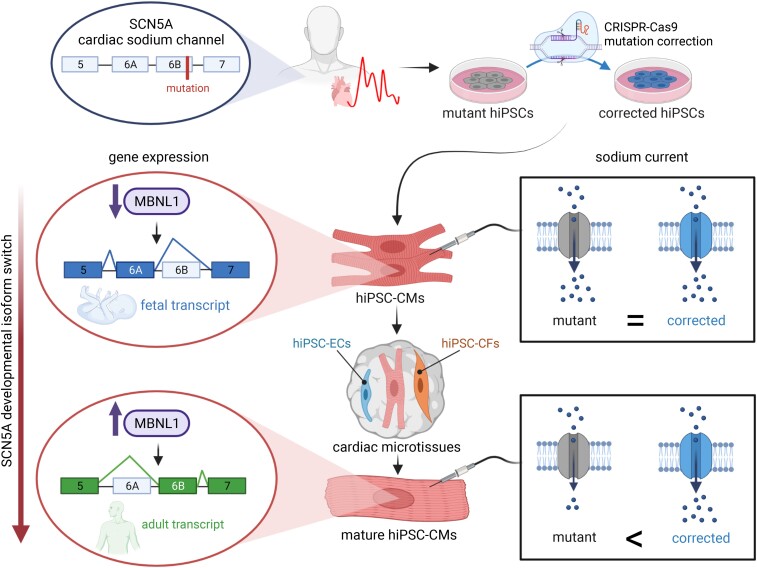
hiPSC-CM maturation in 3D cardiac microtissues promotes SCN5A fetal to adult isoform switch.

## 1. Introduction

Human-induced pluripotent stem cells (hiPSCs) are widely used to derive cardiomyocytes (hiPSC-CMs) and study cardiac diseases at the cellular and molecular level.^1–3^ Patient-specific hiPSC-CMs capture the genetic landscape of the affected individual, allowing precise dissection of pathogenic mechanisms and testing drug responses that may ultimately lead to (personalized) treatments. Among the cardiac diseases that have been modelled using hiPSC-CMs are inherited cardiac arrhythmias caused by mutations in cardiac ion channels.^1,4^ The *SCN5A* gene encodes the pore-forming α-subunit of the cardiac sodium (Na^+^) voltage-gated channel Nav1.5. This channel conducts the depolarizing Na^+^ current (*I*_Na_), which is responsible for the fast upstroke of the action potential (AP) in working cardiomyocytes; it is essential for conducting the electrical impulse in the heart. Mutations in *SCN5A* are associated with different cardiac diseases, including Brugada syndrome, long QT syndrome, isolated conduction defects, and dilated cardiomyopathy.^5^ Nav1.5 expression is developmentally regulated, with foetal - or adult isoforms generated by alternative splicing of the *SCN5A* exon 6 (6A foetal and 6B adult), which encodes the voltage sensor of the first channel subunit.^6^ The mechanism of *SCN5A* alternative splicing regulation and other cardiac genes during development has been studied in animal models, where the muscleblind-like RNA-binding protein 1 (Mbnl1) has been identified as key for inducing a post-natal switch.^7–9^ In humans, MBNL1 is sequestered in the heart of myotonic dystrophy patients causing re-expression of the foetal *SCN5A* exon 6A^10^ and the importance of MBNL proteins for late myogenic maturation has been recently demonstrated using hiPSCs.^11^

hiPSC-CMs can recapitulate many electrical alterations due to mutations in *SCN5A* or in genes of Nav1.5 beta-subunits^12–22^ and these are in some cases notably different from those observed in commonly used heterologous expression systems,^23^ most likely because hiPSC-CMs carry the full complement of cardiac ion channel and accessory protein genes. Moreover, gene editing in hiPSCs allows effects of specific mutations to be distinguished from potential confounding elements resulting from line-to-line variability; patient mutations can be introduced into wild-type (WT) hiPSCs or mutations in patient hiPSC lines can be corrected.^15,24^ However, cardiomyocytes differentiated from hiPSCs have an immature (foetal-like) phenotype with their electrical properties, structure, and gene expression profiles resembling first or second gestational trimester foetal cardiac cells^25,26^ rather than adult cardiomyocytes. Although *I*_Na_ in hiPSC-CMs display half-maximal potential (*V*_1/2_) of activation and inactivation consistent with values reported for native human ventricular myocytes,^27^ the maximal AP upstroke velocity (*V*_max_) is slower than in adult cardiomyocytes.^28^ In addition, hiPSC-CMs mostly express the Nav1.5 foetal isoform, masking effects due to mutations located in the adult exon 6B.^29^ Previously, we and others addressed the issue of *SCN5A in vitro* maturation by culturing hiPSC-CMs for long periods to promote spontaneous maturation^29^ and by using a medium inducing a metabolic switch from glycolysis to fatty acid oxidation.^20^ We recently discovered that hiPSC-CM maturation is overall enhanced in a three-dimensional (3D) microtissue (MT) environment containing cardiac fibroblasts (CFs) and endothelial cells (ECs).^30^ hiPSC-CMs isolated from MTs have more hyperpolarized RMP and faster AP upstroke velocity, suggesting an electrical maturation involving Nav1.5.

Here, we showed that maturation of hiPSC-CMs in MTs promoted expression of the *SCN5A* adult isoform and allowed (i) evaluation of functional effects of the p.R225W mutation, located in adult exon 6B and (ii) detection of the more severe disease phenotype associated with the compound heterozygosity for p.W156X mutation,^13^ as observed in patients.^31^ Using gene-corrected isogenic hiPSCs was crucial in identifying mutation-specific effects. We also found that *MBNL1* up-regulation in MTs was necessary and sufficient to induce the expression of *SCN5A* exon 6B.

## 2. Methods

### 2.1 Patients’ clinical history and phenotype

**Figure 1:**
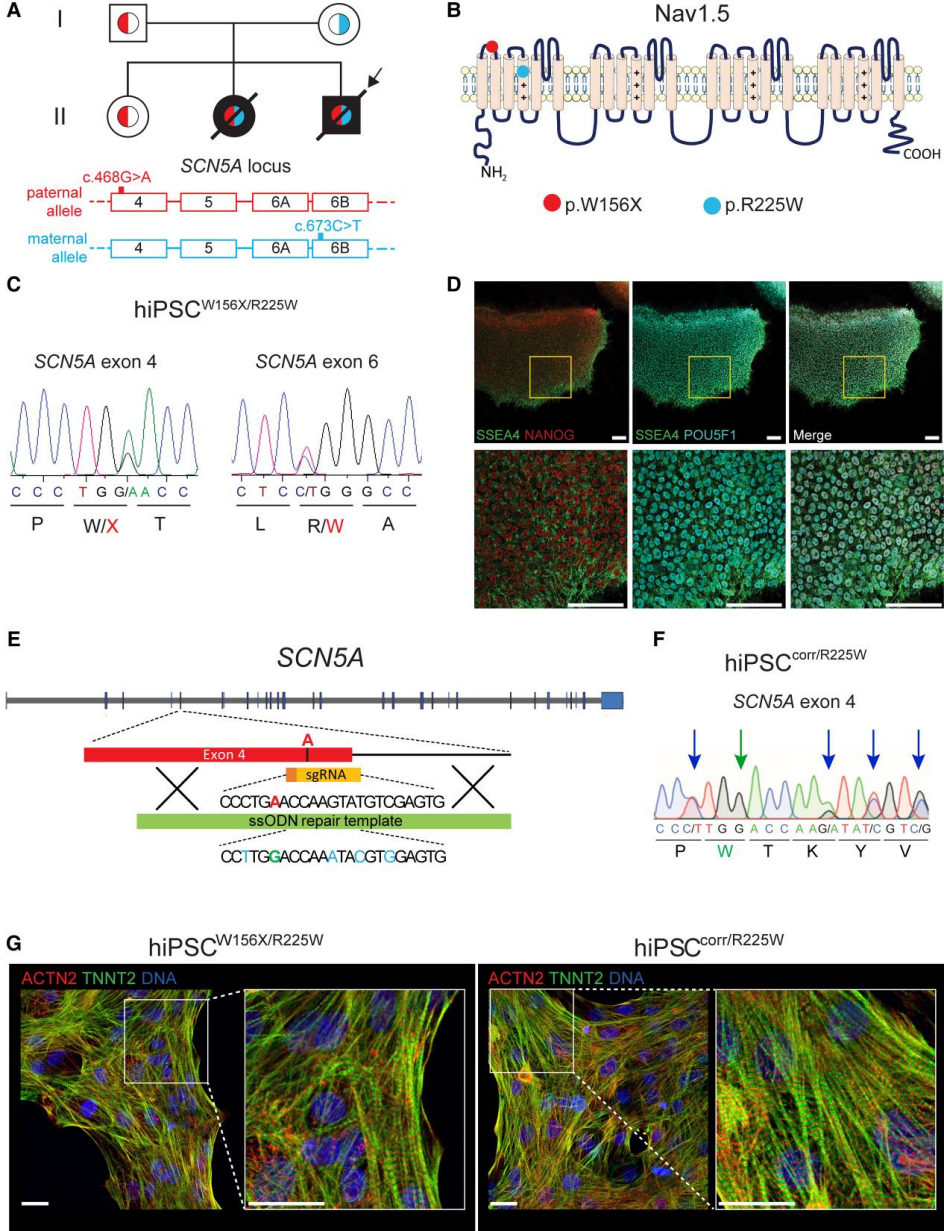
Generation of hiPSCs from a patient with compound heterozygous mutations in *SCN5A* and their genetic correction. (*A*) Top, family tree of the proband (arrow) showing in black filling the patients affected by cardiac conduction defects and in white unaffected individuals. Red (left) and light blue (right) colours within the family tree symbols indicate the genotype at the *SCN5A* locus, as indicated at the bottom of the figure. Red (top), paternal allele carrying the c.468G>A *SCN5A* (p.W156X) mutation in exon 4; light blue (bottom), maternal allele carrying the c.673C>T *SCN5A* (p.R225W) mutation in exon 6B. (*B*) Schematic representation of the sodium channel Nav1.5 α-subunit, encoded by *SCN5A*. The red (left) and the light blue (right) circles show the position of p.W156X and p.R225W mutations, respectively. (*C*) Sanger sequencing chromatograms showing the c.468G>A *SCN5A* (p.W156X) mutation in exon 4 (left), and the c.673C>T *SCN5A* (p.R225W) mutation in exon 6B (right), both present in heterozygosis in the patient-derived hiPSC^W156X/R225W^ line. (*D*) Representative immunofluorescence images of hiPSC^W156X/R225W^ undifferentiated colonies showing expression of pluripotency markers NANOG (red), SSEA4 (green), and POU5F1 (cyan). Bottom panels are an enlargement of the framed area in top panels. Scale bars: 25 µm. (*E*) Schematic showing the strategy used to correct the c.468G>A (p.W156X) mutation in *SCN5A* exon 4 with CRISPR/Cas9 in the paternal allele of hiPSC^W156X/R225W^. The mutant adenine base is shown in red; in yellow the sgRNA guiding the Cas9 to the mutation; in green the ssODN used as donor template for homology-directed DNA repair. Underneath part of the ssODN sequence, showing in green the WT guanine base and in light blue the silent mutations. (*F*) Sanger sequencing chromatogram showing *SCN5A* exon 4 after correction of the c.468G>A (W156X) mutation in hiPSC^corr/R225W^. The green arrow (second arrow from the left) indicates the corrected patient mutation and the blue arrows indicate the silent mutations inserted in one allele. (*G*) Representative immunofluorescence staining for ACTN2 (red) and TNNI3 (green) in hiPSC^W156X/R225W^- and hiPSC^corr/R225W^-CMs. Nuclei are stained with Dapi (blue). Panels on the right are enlargement of the framed area in the left panels. Scale bars: 10 µm.

The clinical characteristics of the family were previously reported.^31^ The proband patient (*Figure 1A*, II-3), born with a severe tachycardia and conduction disorder, was treated with beta-blockers and later fitted with an ICD implant. Despite the ICD, he died from intractable ventricular arrhythmias at age 21. The parents and a sister (*Figure 1A*, II-1) of the proband were asymptomatic, whereas the other sister (*Figure 1A*, II-2) carrying the same mutations of the proband was affected by intractable arrhythmia and died at age of 1.

### 2.2 hiPSCs generation and culture

A skin punch biopsy was obtained following written informed consent from the patient at the age of 18 and approval by the medical ethics committee of the Amsterdam Medical Center (AMC), in accordance with the Declaration of Helsinki. Dermal fibroblasts were reprogrammed using the Sendai virus by the Leiden University Medical Center (LUMC) hiPSC core facility, as described previously,^13^ following the protocol approved by the LUMC and AMC ethical committees. Details for hiPSC culture and differentiation are reported in the Supplementary material online.

### 2.3 hiPSC editing using CRISPR/Cas9

For the correction of c.468G>A mutation in SCN5A exon 4 (W156X) and excision of SCN5A exon 6A specific sgRNA sequences (*Figures 1E* and *3A*, Supplementary material online, *Table S1*) were cloned into the pSpCas9(BB)-2A-Puro (PX459) v2 plasmid encoding Cas9 nuclease and puromycin resistance (Addgene #62988).^32^ For the correction of c.673C > T mutation in exon 6B (p.R225W) and the excision of exon 4 of *MBNL1*, the Alt-R® CRISPR–Cas9 System (IDT Technologies) was used. Specific single-stranded DNA oligonucleotide (ssODN) repair template carrying the WT base and silent mutations were used for each mutation correction (see Supplementary material online, *Table S1*). Additional information is provided in the Supplementary material online.

### 2.4 Formation of 3D cardiac MTs

MTs were formed by combining defined ratios of hiPSC-CMs, -ECs, and -CFs (70:15:15), as previously described.^30,33^

### 2.5 Electrophysiology

Beating monolayers of hiPSC-CMs were dissociated at day 21 of differentiation using Tryple Select 0.5× (ThermoFisher Scientific) and plated onto Matrigel-coated glass coverslips at low density. Electrophysiological properties of hiPSC-CMs were analysed 10 days after dissociation. MTs were dissociated at day 21 of culture using collagenase II (Worthington Industries), as previously described.^33^ Cells were plated on Matrigel-coated coverslips in LI-BPEL supplemented with 50 ng/mL VEGF and 5 ng/mL bFGF, refreshed with LI-BPEL medium the next day, and analysed for electrophysiological properties 7–10 days after dissociation.

Patch-clamp experiments were performed using an Axopatch 200B amplifier (Molecular Devices, Sunnyvale, CA, USA). Clampex 10.7 was used for data acquisition, voltage control, and analysis. APs were recorded in isolated single cells using the perforated patch-clamp technique in current-clamp mode and sodium current (*I*_Na_) was recorded with the ruptured patch-clamp technique in voltage-clamp mode. Patch-clamp solutions and protocols are reported in the Supplementary material online.

### 2.6 RNA isolation, cDNA synthesis, and gene expression analysis

Total RNA was isolated either from hiPSC-CMs on day 21 of differentiation and from hiPSCs using Nucleospin kit (Machery and Nagel), or from MTs on day 21 of culture using RNeasy Micro Kit (Qiagen), according to the manufacturers’ instructions. cDNA was synthesized using iScript™ cDNA Synthesis Kit and gene expression was analysed by qPCR with IQ Syber Green Supermix (Bio-Rad), according to the manufacturer’s protocol. All qPCR reactions were performed in duplicate. Gene expression levels were normalized to the *RPL37A* housekeeping gene.

For quantification of mRNA-splicing isoforms containing 6A or 6B exons, BioRad QX200™ Droplet Digital PCR (ddPCR) system was used following the manufacturer’s instructions. Primer and probe sequences used for qPCR and ddPCR are reported in Supplementary material online, *Table S2 and S3*.

### 2.7 RNA-seq analysis

The RNA was extracted with the RNeasy micro kit (QIAGEN) as from the manufacturer’s instructions, followed by paired-end sequencing at 150 bp, 40 million reads for each sample (Novogene, Cambridge, UK). Residual Illumina adapters were trimmed from the RNA sequences using cutadapt^34^ with parameter -m 20. Reads were aligned to the human genome (build 38, Ensembl version 96) using STAR.^35^ BAM files were used by rMATS^36^ to detect differentially spliced exons and sashimi plots were made using the function rmats2sashimiplot (https://github.com/Xinglab/rmats2sashimiplot).

Exon counts for the SCN5A and MBNL1 canonical transcripts, ENST00000413689 and ENST00000282486, respectively, were retrieved using featureCounts^37^ with parameters -t exon and -g exon_id. Transcripts per kilobase million (TPM) was calculated considering all exons counts as library size and by using the R script convertCounts. The coverage of constitutive and alternative exons for SCN5A or MBNL1 was calculated as percentage of TPM assigned to each exon bin over the TPM mean of the whole transcript.

RNA-seq data are available in the GEO repository with accession number GSE180290. Publicly available foetal heart (FH) and adult heart (AH) RNA-seq data (GEO, accession number GSE62913^38^) were used for Supplementary material online, *Figure S7A*.

### 2.8 *In vitro* generation of MBNL1 mRNA and transfection

MBNL1 mRNA was generated by *in vitro* transcription using INCOGNITO T7 5mC- and Ψ-RNA Transcription Kit, ScriptCap Cap 1 Capping System and A-Plus Poly(A) Polymerase Tailing Kit (all from Cellscript, LLC) following the manufacturer’s instructions.

hiPSC-CMs were transfected with MBNL1 mRNA using Lipofectamine Stem Transfection Reagent (Invitrogen) according to the manufacturer’s instructions. Details are reported in the Supplementary material online.

### 2.9 Statistical analysis

Data were obtained from at least three independent differentiation- or MT formation experiments and were expressed as mean ± standard error of the mean (SEM). Data were compared using *t*-test or one-way ANOVA followed by Fisher LSD *post hoc* test. Statistical analysis was performed using OriginPro 2016 (Origin Lab), RStudio, or GraphPad Prism (GraphPad Software, Inc.).

## 3. Results

### 3.1 Generation and genetic correction of hiPSCs from a patient with compound heterozygous mutations in *SCN5A*

A patient (*Figure 1A*, II-3) presented with severe cardiac conduction abnormalities and ventricular arrhythmias early after birth and was genetically characterized as carrying bi-allelic mutations in the *SCN5A* gene.^31^ These were a c.468G>A nucleotide change in exon 4 of *SCN5A* (NM_198056.2) inherited from the father, leading to the stop codon mutation p.W156X (NP_932173.1), and a c.673C>T nucleotide change in exon 6B of *SCN5A* inherited from the mother. This led to the substitution of a positively charged arginine with an aromatic tryptophan (p.R225W) in the voltage-sensor segment of the first domain of the channel (*Figure 1A* and *B*). When the patient turned 18, we reprogrammed dermal skin fibroblasts using the Sendai virus to generate the hiPSC^W156X/R225W^ line. Sanger sequencing confirmed the presence of both *SCN5A* mutations (*Figure 1C*). After several passages, hiPSCs were negative for the Sendai virus (see Supplementary material online, Figure*S1A*), had a normal karyotype (see Supplementary material online, *Figure S1B*), expressed the pluripotency markers NANOG, SSEA4, POU5F1 (*Figure 1D*), and genome-wide gene expression analysis indicated high ‘pluripotency score’ and low ‘novelty score’ with the PluriTest algorithm^39^ (see Supplementary material online, *Figure S1C*, left).

While the p.W156X Nav1.5 mutation was previously characterized in a heterologous system^31^ and using hiPSC-CMs derived from the proband’s father (*Figure 1A*, I-1),^13^ the phenotypic effects of the p.R255W Nav1.5 mutation was only studied in heterologous system^31^ since hiPSCs from the proband’s mother were not available. To examine the effects of this mutation specifically, we corrected the c.468G>A *SCN5A* mutation in the hiPSC^W156X/R225W^ line using CRISPR–Cas9 and generated the hiPSC^corr/R225W^ line (*Figure 1E* and *F*). One hiPSC clone was selected, in which Sanger sequencing confirmed the correction of the c.468G>A *SCN5A* mutation and the presence of the silent mutations in heterozygosity (*Figure 1F*). No modifications were present in the *in silico*-predicted two intragenic Cas9 off-target sites by Sanger sequencing (data not shown). hiPSC^corr/R225W^ had a normal karyotype (see Supplementary material online, *Figure S1D*) and high pluripotency score, as confirmed by PluriTest (see Supplementary material online, *Figure S1C*, right). The original hiPSC^W156X/R225W^ line was heterozygous for the rs6781731 SNP located in the intron downstream exon 4. The corrected hiPSC^corr/R225W^ was also heterozygous for this SNP, confirming that the recombination involved only the paternal allele as intended (see Supplementary material online, *Figure S1E*).

### 3.2 No functional effects of exon 6B mutation in monolayer hiPSC-CM cultures expressing the foetal but not adult *SCN5A* isoform

We differentiated hiPSC^W156X/R225W^ and hiPSC^corr/R225W^ lines into cardiomyocytes (CMs) in monolayer (*Figure 1G*). Differentiation efficiencies were comparable between the two lines and consistently yielded >90% cardiac troponin T (TNNT2)-positive cells (see Supplementary material online, Figure*S2A*). The cardiac ion channel genes *SCN5A*, *NCX1*, *KCND2*, *KCNH2*, *KCNQ1*, and *KCNJ12* were expressed at similar levels in CMs from hiPSC^W156X/R225W^ and hiPSC^corr/R225W^ (see Supplementary material online, Figure*S2B*).

**Figure 2:**
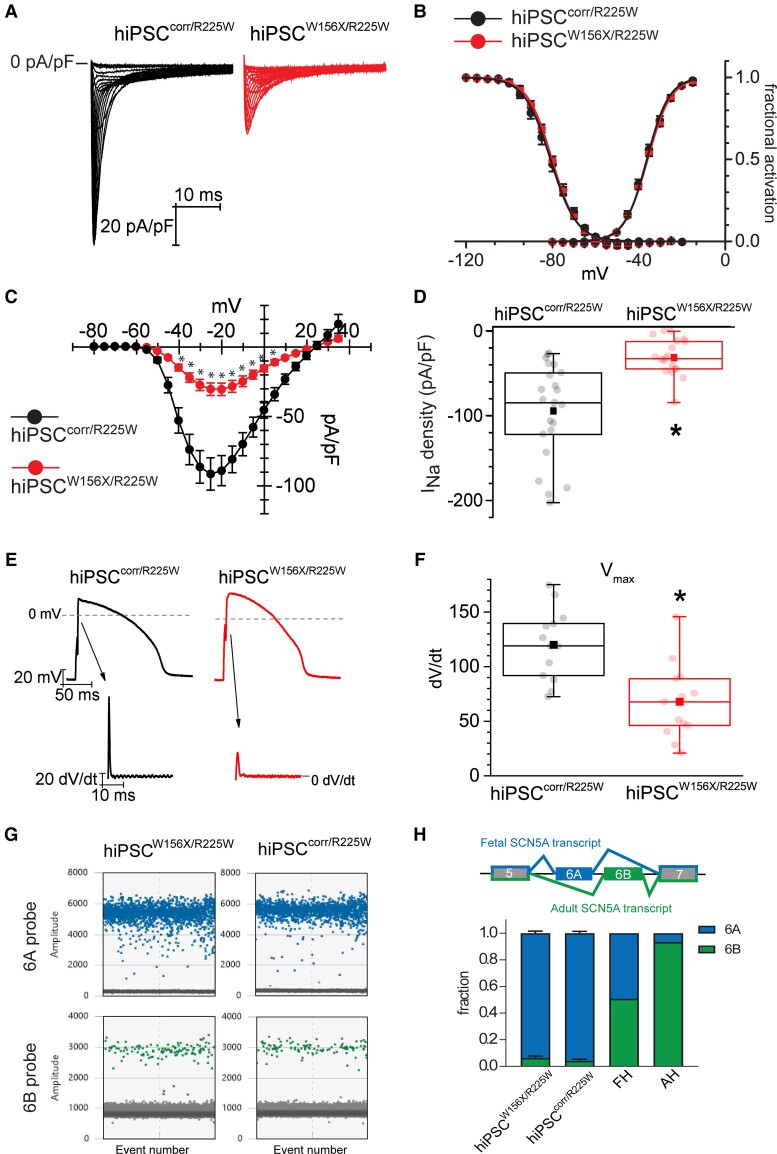
Cardiomyocytes from hiPSCW^156X/R225W^ and hiPSC^corr/R225W^ show no altered electrical properties due to exon 6B-located p.R225W SCN5A mutation and express mainly the foetal *SCN5A* isoform. (*A*) Representative *I*_Na_ traces recorded as indicated in hiPSC^corr/R225W^- (black) and hiPSC^W156X/R225W^- (red) CMs during voltage-clamp activation protocol (test range −80/−15 mV, 22 steps, holding potential = −100 mV). (*B*) Average activation (AC) and inactivation (IC) curve of *I*_Na_ recorded in hiPSC^corr/R225W^- (black; AC: *V*_1/2_ = −36.3 ± 0.7 mV, *n* = 21; IC: *V*_1/2_ = −80.9 ± 1 .2 mV, *n* = 15) and hiPSC^W156X/R225W^- (red; AC: *V*_1/2_ = −35.9 ± 0.4 mV, *n* = 17; IC: *V*_1/2_ = −80.2 ± 0.7 mV, *n* = 14) CMs. *P* > 0.05 with Student’s *t*-test. (*C*) Mean current–voltage (*I*–*V*) relationships of *I*_Na_ recorded in hiPSC^corr/R225W^ (black, *n* = 23) and hiPSC^W156X/R225W^ (red, *n* = 21) CMs, showing a reduction in current density in hiPSC^W156X/R225W^. Experiments >4. *P < 0.05 with two-way ANOVA repeated measures. Data in (*B*) and (*C*) are shown as mean ± SEM. (*D*) Box plot of maximal peak *I*_Na_ density in hiPSC^corr/R225W^ (black) and hiPSC^W156X/R225W^ (red) CMs. Dots: single values, lines: median, square: mean, error bars: 1.5× inter-quartile range (IQR). *P < 0.05 with Student’s *t*-test. (*E*) Representative APs recorded from hiPSC^corr/R225W^- (black) and hiPSC^W156X/R225W^- (red) CMs paced at 1 Hz. Arrows indicate the respective derivative trace of the AP upstroke, showing a smaller peak in hiPSC^W156X/R225W^ which corresponds to a slower *V*_max_. (*F*) Box plot of mean *V*_max_ of APs recorded in hiPSC^corr/R225W^- (black) and hiPSC^W156X/R225W^- (red) CMs. Dots, lines, squares and error bars as in (*D*). *n* = 13, experiments = 4. *P < 0.05 with Student’s *t*-test. (*G*) Representative outcome of the ddPCR assay showing exon 6A (blue) and 6B (green) expression in hiPSC^W156X/R225W^- and hiPSC^corr/R225W^-CMs. Each coloured dot represents a positive droplet for the fluorophore, grey dots represent negative droplets. (*H*) Top, schematic of the developmentally regulated alternative splicing of *SCN5A* exon 6: the foetal *SCN5A* transcript includes exon 6A, whereas the adult *SCN5A* transcript includes exon 6B. Bottom, bar graph showing the average fraction of *SCN5A* exon 6A (blue) and 6B (green) expression in 20-day-old hiPSC^W156X/R225W^- (*n* = 5) and hiPSC^corr/^R225W-CMs (*n* = 6) compared with foetal heart (FH) and adult heart (AH).

We next examined the electrophysiological properties of CMs from hiPSC^W156X/R225W^ and hiPSC^corr/R225W^ lines. *Figure 2A* shows representative *I*_Na_ traces from hiPSC^W156X/R225W^- and hiPSC^corr/R225W^-CMs. While the voltage dependence of *I*_Na_ activation and inactivation was similar between the two lines (see Supplementary material online, *Figure 2B*), *I*_Na_ density was lower in hiPSC^W156X/R225W^-CMs over the whole physiological voltage range (*Figure 2C*), with the mean peak current density in hiPSC^W156X/R225W^-CMs (−31 ± 4.5 pA/pF) significantly reduced compared with hiPSC^corr/R225W^-CMs (−94.2 ± 11.4 pA/pF) (*Figure 2D*). Notably, the current density recorded in hiPSC^corr/R225W^-CMs was comparable to that previously reported (∼ −100 pA/pF) for CMs differentiated with the same method from a WT hiPSC line (hiPSC^WT/WT^), whereas *I*_Na_ density in hiPSC^W156X/R225W^-CMs was similar to that recorded in hiPSC-CMs carrying only the p.W156X mutation (∼ −30 pA/pF) derived from the proband’s father (*Figure 1A* I-1).^13^

Altered *I*_Na_ impacts the electrical activity of cardiomyocytes, particularly the AP upstroke velocity. We, therefore, recorded APs in hiPSC^W156X/R225W^- and hiPSC^corr/R225W^-CMs paced at 1 Hz. To compensate for the typical depolarized RMP of foetal-like hiPSC-CMs, we used dynamic clamp to set the RMP to −86 mV.^40^*Figure 2E* shows representative AP traces recorded from hiPSC^W156X/R225W^- and hiPSC^corr/R225W^-CMs, and their respective first derivative trace. In agreement with the differences in *I*_Na_ density, the maximal first derivative of AP upstroke, corresponding to the maximal upstroke velocity (*V*_max_), was significantly lower in hiPSC^W156X/R225W^- compared with hiPSC^corr/R225W^-CMs (*Figure 2F*). As observed for *I*_Na_, *V*_max_ values from hiPSC^corr/R225W^-CMs were similar to those previously reported for WT hiPSC-CMs using the same dynamic clamp technique.^41^ Other AP parameters, namely RMP, AP amplitude (APA), and AP duration (APD), were not different between the two lines (see Supplementary material online, Figure*S2C*).

To check whether both mutations contributed to *I*_Na_ and *V*_max_, we measured exon 6A and 6B expression in CMs from hiPSC^W156X/R225W^ and hiPSC^corr/R225W^ lines. Using digital droplet PCR (ddPCR) we quantified exon 6A- and exon 6B-containing transcripts (see Supplementary material online, Figure*S2D and E*). *Figure 2G* illustrates representative ddPCR results for hiPSC^W156X/R225W^- and hiPSC^corr/R225W^-CMs. Exon 6B-containing *SCN5A* transcripts accounted for approximately 5% and were much lower than exon 6A-containing transcripts (*Figure 2G* and *H*). In FH and AH tissues, exon 6B-containing transcripts accounted for 50% and 94% of all transcripts, respectively, in line with previous reports,^29,42^ and thus were expressed at higher levels than in hiPSC-CMs (*Figure 2H*).

These results indicated that hiPSC^W156X/R225W^ and hiPSC^corr/R225W^-CMs expressed the foetal *SCN5A* isoform almost exclusively and *I*_Na_ density and *V*_max_ reduction in the compound mutant was due to the p.W156X Nav1.5 mutation. The effect of p.R225W mutation could not be properly determined.

### 3.3 Genetic excision of exon 6A does not increase adult *SCN5A* isoform expression

**Figure 3:**
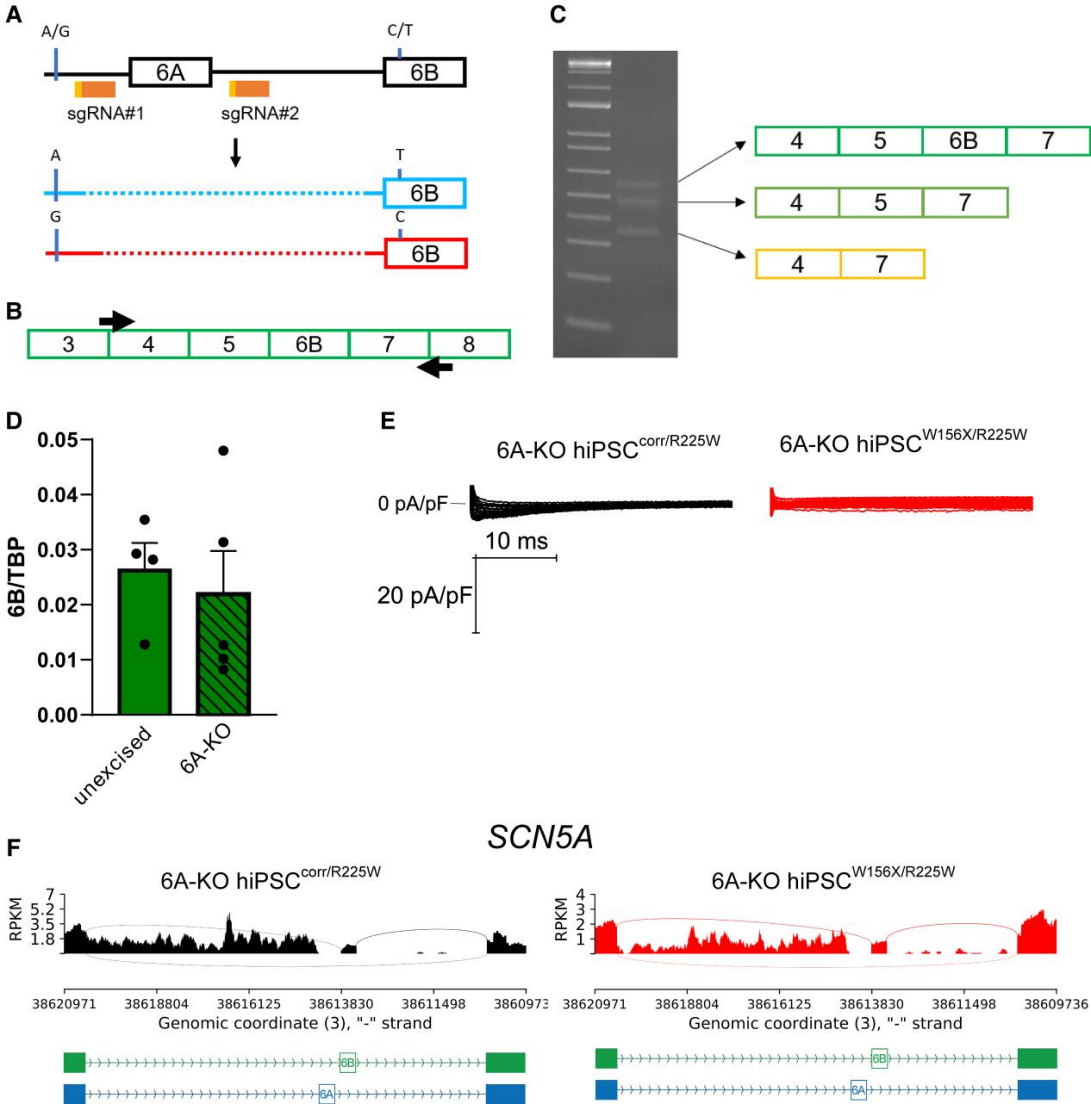
Genetic excision of exon 6A does not increase adult *SCN5A* isoform expression. (*A*) Schematic representation of the strategy to excise *SCN5A* exon 6A using CRISPR/Cas9 with two sgRNAs (sgRNA#1 and #2, orange). In red and light blue, the results of excision (dotted line) in the hiPSCs^W156X/R225W^: the maternal allele (top, blue, with the mutated T in exon 6B and the A SNP in the intron between exon 5 and exon 6A) carried a 15 bp larger deletion upstream of exon 6A compared with the paternal allele (bottom, red, WT C in exon 6B and G SNP in 5-6A intron). (*B*) Schematic showing the primers (black arrows) used to amplify and sequencing cDNA from 6A-KO hiPSC^corr/R225W^-CMs. (*C*) Gel electrophoresis of PCR products from (*B*) showing three bands corresponding to three transcript species as indicated by the arrows. (*D*) Bar graph of mean expression of exon 6B in unexcised and 6A-KO hiPSC-CMs. Data were normalized to *TBP*. *n* = 4. Dots: single values. (*E*) Representative *I*_Na_ traces recorded from 6A-KO hiPSC^corr/R225W^ and 6A-KO hiPSC^W156X/R225W^ CMs showing an almost complete absence of the current. (*F*) Sashimi plots of RNA-seq data from 6A-KO hiPSC^corr/R225W^ and 6A-KO hiPSC^W156X/R225W^ CMs showing the absence of exon 6A transcription but extensive transcription of the intronic region between exon 5 and exon 6B.

To investigate whether the removal of exon 6A might force *SCN5A* exon 6B expression and thus reveal the effect of the p.R225W Nav1.5 mutation, we used CRISPR–Cas9 to excise this exon from both *SCN5A* alleles in the hiPSC^W156X/R225W^ line. As illustrated in *Figure 3A*, we designed 2 gRNAs targeted to the flanking regions of exon 6A and identified a clone in which exon 6A had been excised in both alleles. The actual excision spanned a larger intronic region than intended by the strategy design (*Figure 3A* and Supplementary material online, *S3A*). In the hiPSC lines with complete exon 6A excision (referred to as 6A-KO hiPSC^W156X/R225W^), we corrected the p.W156X mutation in exon 4 (generating the 6A-KO hiPSC^corr/R225W^ line), using CRISPR–Cas9 with the same strategy as earlier (*Figure 1E*). The excised hiPSC lines differentiated into CMs with similar efficiencies and beating properties as the parental hiPSC line (data not shown). As expected, exon 6A was not expressed at all in excised CMs at 21 or 60 days of culture, whereas expression of exon 6B was detected (see Supplementary material online, Figure*S3B*). To examine *SCN5A* transcripts expressed by CMs from the excised hiPSC lines, we amplified by PCR a region between exon 3 and exon 8 of *SCN5A* (*Figure 3B*). Here, we only used the 6A-KO hiPSC^corr/R225W^ line, since in the 6A-KO hiPSC^W156X/R225W^ line, the stop codon p.W156X mutation in exon 4 leads to nonsense-mediated mRNA decay^13^ and could confound the results. Gel electrophoresis of PCR products revealed three distinct bands (*Figure 3C*). Sanger sequencing demonstrated these corresponded to three different transcript species: the highest band represented the adult isoform of *SCN5A* transcript including exon 6B; the middle band, a transcript in which exon 6B was skipped, and in the lowest band, both exons 5 and 6B were skipped (*Figure 3C*).

To investigate whether the overall expression of the adult *SCN5A* isoform was increased compared with the non-excised line, we performed ddPCR analysis, normalizing the expression of exon 6B with that of the TATA-binding protein (TBP). Exon 6B expression was similar in the excised hiPSC-CMs and in the parental non-excised hiPSC-CMs (*Figure 3D*). Since exon 6B-containing transcripts were likely the only functional transcript species produced in the exon 6A-excised lines, we investigated whether this would unmask the functional phenotype of the p.R225W Nav1.5 with electrophysiology. We attempted to record *I*_Na_ in 6A-KO hiPSC^W156X/R225W^- and 6A-KO hiPSC^corr/R225W^-CMs but *I*_Na_ was virtually absent in all (*n* = 9) 6A-KO hiPSC^W156X/R225W^-CMs (*Figure 3E*), while 50% of the 6A-KO hiPSC^corr/R225W^ cells (*n* = 10) showed very small *I*_Na_ (*Figure 3E*) with mean peak current density of −12.9 ± 5.5 pA/pF. Analysis of *SCN5A* transcript expression by RNA-seq showed that upon excision of exon 6A, not only was there spurious mRNA produced but also the intronic region between exon 5 and exon 6B was aberrantly included at a similar level to the exons (*Figure 3F*).

Taken together, these results demonstrated that excision of *SCN5A* exon 6A impairs the splicing mechanism, leading to abnormally spliced transcripts of unknown significance and the inclusion of intronic regions in the transcript population. Importantly, 6B-containing transcript levels were not sufficient to generate detectable *I*_Na_ for comparison of the two mutant lines.

### 3.4 Electrical maturation of hiPSC-CMs in 3D cardiac MTs with hiPSC-derived cardiac non-myocytes reveals functional effects of p.R225W mutant Nav1.5

**Figure 4:**
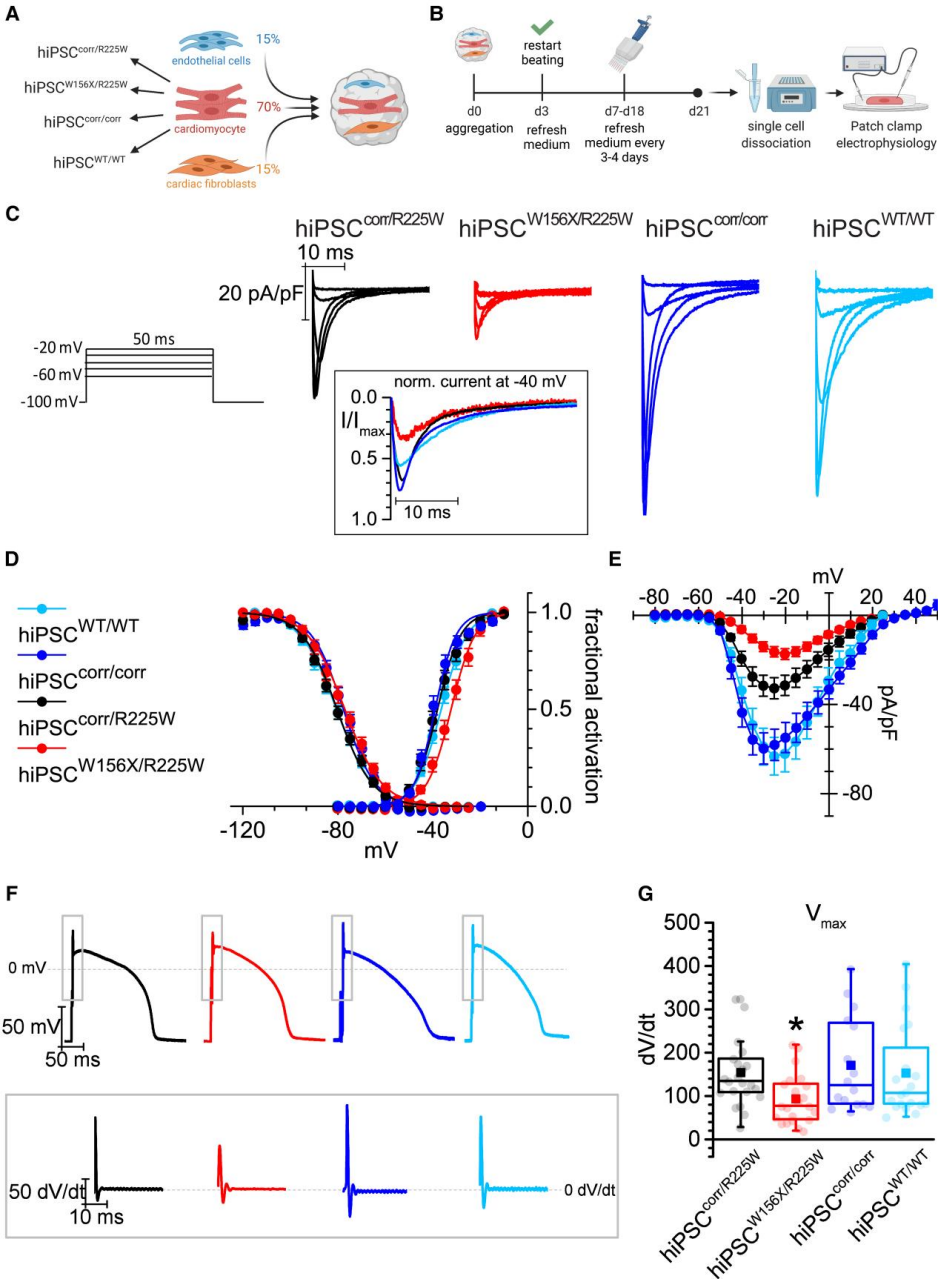
Electrical maturation of hiPSC-CMs in 3D cardiac microtissues with hiPSC-derived non-myocytes reveals functional effects of p.R225W SCN5A mutation. (*A*) Schematic of the microtissue formation using hiPSC-CMs from four lines (hiPSC^corr/R225W^, hiPSC^W156X/R225W^, hiPSC^corr/corr^, hiPSC^WT/WT^) and ECs and CFs from hiPSC^WT/WT^. The percentage of each cell type is indicated. (*B*) Schematic of 21-day MT culture protocol until single-cell dissociation for analysis. (*C*) Representative *I*_Na_ traces recorded in hiPSC^corr/R225W^-, hiPSC^W156X/R225W^-, hiPSC^corr/corr^-, and hiPSC^WT/WT^-CMs dissociated from MTs, corresponding to the voltage steps reported on the right. In the inset, the current at −40 mV normalized to the peak current, to compare the fraction of open channels around the *V*_1/2_ of activation. (*D*) Activation (AC) and inactivation (IC) curve of *I*_Na_ recorded in hiPSC^corr/R225W^- (black; AC: *V*_1/2_ = −38.1 ± 1.2 mV; IC: *V*_1/2_ = −80.6 ± 1.1 mV; *n* = 16), hiPSC^W156X/R225W^- (red; AC: *V*_1/2_ = −32.4 ± 1.2 mV*, *n* = 11; IC: *V*_1/2_ = −77.3 ± 1.4 mV, *n* = 9), hiPSC^corr/corr^- (blue; AC: *V*_1/2_ = −39.3 ± 0.9 mV, *n* = 17; IC: *V*_1/2_ = −77.9 ± 1.2 mV; *n* = 16), and hiPSC^WT/WT^- (light blue; AC: *V*_1/2_ = −36.5 ± 0.9 mV, *n* = 8; IC: *V*_1/2_ = −80.8 ± 1.3 mV, *n* = 7) CMs showing a rightward shift in *I*_Na_ activation in hiPSC^W156X/R225W^-CMs. *P < 0.05 vs. the other lines with one-way ANOVA and Fisher’s *post hoc* test. Experiments = 3. (*E*) Mean voltage-density plot of *I*_Na_ recorded in hiPSC^corr/R225W^- (black), hiPSC^W156X/R225W^- (red), hiPSC^corr/corr^- (blue), and hiPSC^WT/WT^- (light blue) CMs, showing a reduction in current density in hiPSC^W156X/R225W^- and hiPSC^corr/R225W^-CMs compared with the corrected and WT line and a shift in the activation of hiPSC^W156X/R225W^-CMs. From −45 to −20 mV: *P* < 0.05 for hiPSC^W156X/R225W^ and for hiPSC^corr/corr^ vs. the other lines with two-way ANOVA repeated measures. Experiments = 3. (*F*) Representative AP traces recorded with dynamic clamp from hiPSC^corr/R225W^- (black), hiPSC^W156X/R225W^- (red), hiPSC^corr/corr^- (blue), and hiPSC^WT/WT^- (light blue) CMs paced at 1 Hz. The inset indicates the respective derivative trace of the AP after the stimulus. (*G*) Box plot of mean *V*_max_ of APs recorded in hiPSC^corr/R225W^- (black, *n* = 23), hiPSC^W156X/R225W^- (red, *n* = 22), hiPSC^corr/corr^- (blue, *n* = 17), and hiPSC^WT/WT^- (light blue, *n* = 20) CMs. Dots: single values, lines: median, squares: mean, error bars: 1.5× IQR. *P < 0.05 vs. the other lines with one-way ANOVA and Fisher’s *post hoc* test. Experiments = 4.

We next tested whether hiPSC-CM maturation in cardiac MTs containing hiPSC-derived CFs and cardiac ECs,^30,33^ could promote *SCN5A* exon 6B expression and thus reveal the effect of the p.R225W mutation. The cardiac MTs were generated using defined ratios of the three cell types as shown in *Figure 4A* and dissociated into single cells after 21 days of culture to analyse *I*_Na_ by patch clamp (*Figure 4B*). Since *I*_Na_ had not been characterized in hiPSC-CMs derived in this way before, we used hiPSC-CMs from hiPSC^WT/WT^ as a process control.^13,43^ In addition, we corrected the p.R225W mutation in hiPSC^corr/R225W^, generating the isogenic control hiPSC^corr/corr^ (see Supplementary material online, Figure*S4A*). Correction of the mutation in hiPSC^corr/corr^ was confirmed by Sanger sequencing (see Supplementary material online, Figure*S4B*). The line had a normal karyotype and differentiated efficiently into cardiomyocytes (see Supplementary material online, Figure*S4C* and *D*). Four groups of MTs were analysed, containing CMs from hiPSC^W156X/R225W^, hiPSC^corr/R225W^, hiPSC^corr/corr^ (Supplementary material online, videos S1–S3), and hiPSC^WT/WT^. Representative traces of normalized *I*_Na_ recorded in CMs dissociated from MTs are shown in *Figure 4C*. The inset shows respective current trace at −40 mV normalized on peak current, showing smaller activation for hiPSC^W156X/R225W^ compared with the other lines, indicating that a smaller fraction of *I*_Na_ channels opened at the given voltage. Indeed, the activation curve of hiPSC^W156X/R225W^ showed a significant rightward shift compared with the other lines (*Figure 4D*). *I*_Na_ current density showed instead a genotype-specific reduction, with *I*_Na_ significantly lower along the physiological voltage range in hiPSC^W156X/R225W^-CMs compared with all the other lines, and significantly lower in hiPSC^corr/R225W^- compared with hiPSC^corr/corr^ and hiPSC^WT/WT^-CMs (*Figure 4E*).

Finally, we examined the effect of the altered *I*_Na_ on the AP. *Figure 4F* shows representative AP traces and the derivative of the AP upstroke recorded in hiPSC-CMs dissociated from MTs and paced at 1 Hz. *V*_max_ was significantly smaller in hiPSC^W156X/R225W^-CMs compared with CMs from the other lines (*Figure 4G*), showing that the compound mutations have an additive effect on *I*_Na_ density, thus impacting on CM electrical activity. Other AP parameters such as spontaneous resting membrane potential, APA and APD were instead similar in the different lines (see Supplementary material online, Figure*S5*).

### 3.5 Alternative adult *SCN5A* splicing is promoted in hiPSC-CMs exposed to the MT microenvironment

**Figure 5:**
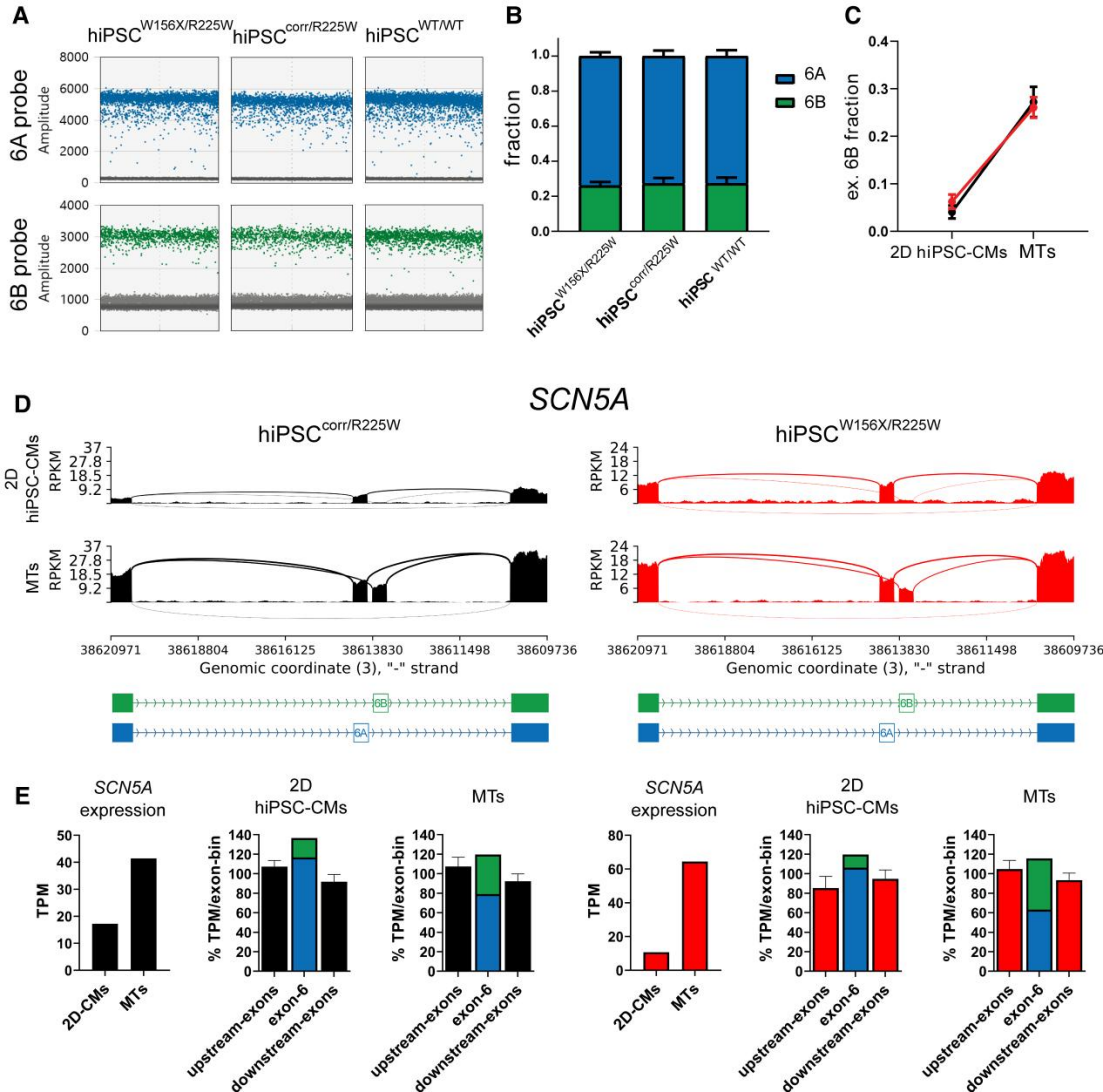
SCN5A exon 6B expression is increased in MTs compared with monolayer hiPSC-CMs. (*A*) Representative outcome of the ddPCR assay showing exon 6A (blue) and 6B (green) expression in hiPSC^W156X/R225W^-, hiPSC^corr/R225W^-, and hiPSC^WT/WT^-CMs. (*B*) Bar graph of the average fraction of *SCN5A* exon 6A and 6B expression in hiPSC^W156X/R225W^-, hiPSC^corr/R225W^- and hiPSC^WT/WT^-MTs (*n* = 3). (*C*) Plot showing the fraction of *SCN5A* exon 6B expression in monolayer (2D) hiPSC-CMs compared with MTs for hiPSC^corr/R225W^ (black) and hiPSC^W156X/R225W^ (red), indicating a strong increase of the relative expression of exon 6B in MTs. (*D*) Sashimi plots of RNA-seq data from 2D hiPSC-CMs and MTs for hiPSC^corr/R225W^ and hiPSC^W156X/R225W^ showing increased expression of *SCN5A* exon 6B in MTs in both lines. (*E*) Bar graphs from RNA-seq data (TPM) from hiPSC^corr/R225W^ (left, black) and hiPSC^W156X/R225W^ (right, red) showing increased expression of *SCN5A* and higher fraction of exon 6B-including transcripts in MTs compared with 2D hiPSC-CMs.

To obtain a more general overview of gene expression, we performed bulk RNA sequencing in hiPSC^W156X/R225W^- and hiPSC^corr/R225W^-2D CMs and 3D MTs after 21 days of culture. Gene ontology (GO) analysis showed that genes related to GO terms of heart development and contraction had a similar expression in the two hiPSC lines, both in 2D CMs and in MTs (see Supplementary material online, Figure*S6A*), indicating that the cells had reached similar levels of differentiation and maturation. Our single-cell (sc) RNA-seq datasets from MTs and hiPSC-CMs previously published from the same WT line used here confirmed that *SCN5A* is only expressed in the hiPSC-CM population (Supplementary material online, *Figure S6B*).^30^ Analysis by ddPCR showed that MTs from hiPSC^W156X/R225W^, hiPSC^corr/R225W^, and hiPSC^WT/WT^ expressed similar fractions of *SCN5A* exon 6B (*Figure 5A*), which accounted for around 27% of total *SCN5A* transcripts (*Figure 5B*). Notably, the fraction of exon 6B-containing transcripts was 5–6 times higher in MTs from hiPSC^W156X/R225W^ and hiPSC^corr/R225W^ compared with their respective hiPSC-CMs derived in monolayer (*Figure 5C*). RNA-seq analysis confirmed that exon 6B expression was increased in MTs compared with monolayer hiPSC-CMs in both lines (*Figure 5D*). Overall *SCN5A* expression was also higher in MTs compared with monolayer hiPSC-CMs in both lines (*Figure 5E*), in agreement with scRNA-seq in hiPSC^WT/WT^ (see Supplementary material online, Figure*S6C*) and confirmed by qPCR (see Supplementary material online, Figure*S6D*, left). The total expression of exon 6 (6A+6B) was similar to upstream and downstream exons; however, the fraction of exon 6B in MTs was higher than in monolayer hiPSC-CMs in both lines (*Figure 5E*). Exon 6B fraction was nonetheless lower in MTs compared with FH and AH (see Supplementary material online, Figure*S7A*). Interestingly, *SCN5A* expression was not increased in MTs containing 6A-KO hiPSC-CMs and the expression of exons downstream of exon 6B was drastically reduced, confirming that 6A excision impaired splicing in the entire gene (see Supplementary material online, Figure*S7B*).

### 3.6 The alternative splicing regulator MBNL1 promotes *SCN5A* exon 6B inclusion during hiPSC-CM maturation

The RNA-binding protein Mbnl1 has been previously shown to promote exon 6B- and inhibit exon 6A expression in the mouse heart.^7,9^ We, therefore, investigated whether MBNL1 is involved in hiPSC-CM alternative splicing.

**Figure 6:**
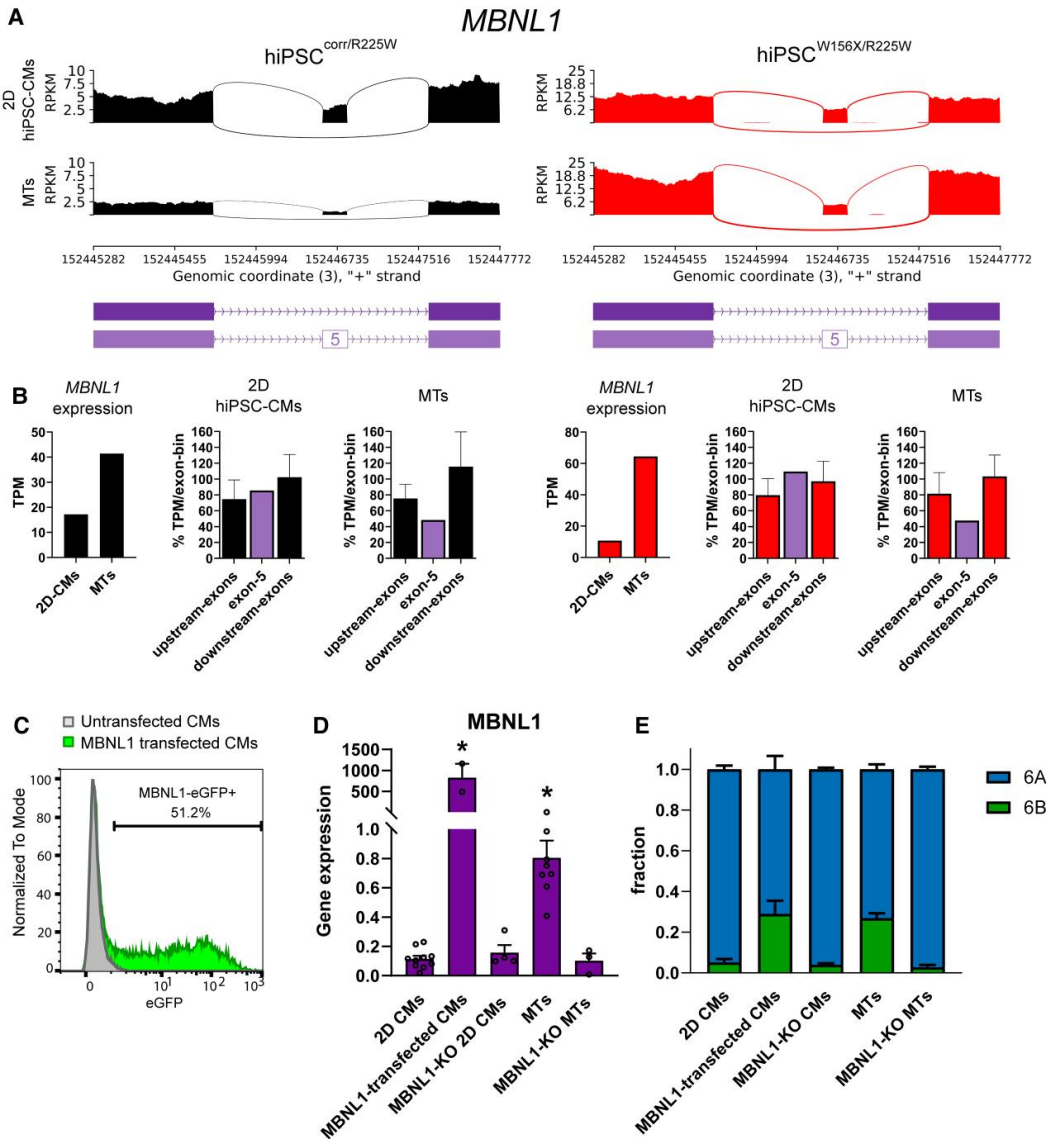
*MBNL1* is up-regulated in MTs and promotes exon 6B expression. (*A*) Sashimi plots of RNA-seq data from 2D hiPSC-CMs and MTs for hiPSC^corr/R225W^ and hiPSC^W156X/R225W^ showing decreased expression of *MBNL1* exon 6 in MTs in both lines. (*B*) Bar graphs showing expression based on RNA-seq data (TPM) from 2D CMs and MTs from hiPSC^corr/R225W^ (left, black) and hiPSC^W156X/R225W^ (right, red); *MBNL1* was up-regulated and a lower fraction of exon 5-including transcripts (purple) in MTs compared with 2D hiPSC-CMs. (*C*) FACS analysis showing the percentage of eGFP-expressing cells (MBNL1-eGFP+) in untransfected (grey) and *MBNL1*-transfected (green) hiPSC-CMs. (*D*) *MBNL1* expression analysis by qPCR in 2D CMs, *MBNL1*-transfected CMs, MBNL1-KO 2D CMs, MTs, and MBNL1-KO MTs. *P < 0.05, One-way ANOVA compared with 2D CMs. *n* > 3. Dots: single values. (*E*) Fraction of exon 6A (blue) and exon 6B (green) *SCN5A* analysed by ddPCR in the cells from (*C*), as indicated. *n* > 3.

RNA-seq analysis showed that *MBNL1* was up-regulated in MTs assembled using hiPSC^W156X/R225W^- and hiPSC^corr/R225W^-CMs compared with 2D hiPSC-CMs cultures (*Figure 6A and B*). Although we found MBNL2 was also up-regulated in MTs (see Supplementary material online, Figure*S6D*), scRNA-seq from our dataset previously published showed that *MBNL1* but not *MBNL2* was up-regulated in the CM population of MTs compared with monolayer hiPSC-CMs (see Supplementary material online, Figure*S6C*), suggesting a major role of MBNL1 specifically in CMs. In addition, MTs displayed skipping of *MBNL1* exon 5, which is promoted by MBNL1 itself in adult CMs^44^ (*Figure 6B*) and thus suggesting that MBNL1 is functional in MTs.

To test whether MBNL1 is necessary and sufficient to promote exon 6B inclusion, we overexpressed or knocked out *MBNL1* in hiPSC-CMs. First, we transiently transfected hiPSC-CMs with an mRNA construct encoding *MBNL1* and eGFP. Transfection efficiency reached around 50%, as shown by FACS analysis of eGFP+ cells (*Figure 6C*) and overexpression of *MBNL1* in transfected hiPSC-CMs compared with untransfected cells was confirmed by qPCR (*Figure 6D*). ddPCR showed that the exon 6B fraction was increased in MBNL1-transfected hiPSC-CMs to levels comparable with those in MTs (*Figure 6E*). We then knocked out *MBNL1* in hiPSCs (*MBNL1*-KO hiPSCs) (see Supplementary material online, Figure*S8*). Cardiac *in vitro* differentiation was not affected in *MBNL1*-KO hiPSCs, but *MBNL1* expression in 2D hiPSC-CMs was negligible and did not differ between unexcised and MBNL1-KO lines (*Figure 6D*). When included in MTs, *MBNL1*-KO hiPSC-CMs did not up-regulate *MBNL1*, differently from the unexcised line (*Figure 6D*). Moreover, exon 6B fraction in MBNL1-KO MTs was comparable to the 2D cultures by ddPCR analysis, and significantly lower than in MTs composed by unexcised hiPSC-CMs (*Figure 6E*).

Taken together these results demonstrated that 3D cardiac MT constructs composed of hiPSC-cardiac ECs and -CFs promoted alternative splicing maturation in hiPSC-CMs such that they displayed increased expression of *SCN5A* exon 6B and its regulator *MBNL1*. This was sufficient to reveal the functional defects caused by the p.R225W mutation on *I*_Na_ and to dissect the different contributions of p.W156X and p.R225W Nav1.5 mutations to the disease in the patient.

## 4. Discussion

The mammalian heart undergoes extensive functional modification after birth, regulated by changes in gene expression at both transcriptional and post-transcriptional level.^45^ Alternative splicing is an important mechanism in the post-natal transition and applies to many cardiac genes,^8,46^ including the cardiac sodium channel Nav1.5.^6,29^ The switch between the foetal *SCN5A* isoform containing exon 6A and the adult *SCN5A* isoform containing exon 6B is completed postnatally in mice and humans.^42,47^ Immature hiPSC-CMs predominantly express the foetal *SCN5A* transcript. Although this does not preclude evaluating the functional effects of *SCN5A* mutations using hiPSC-CMs in general, it does represent a hurdle for those mutations located in exon 6B which is expressed only postnatally. Here, we studied a patient who developed severe cardiac conduction defects early after birth and carried compound mutations in Nav1.5 (p.W156X and p.R225W), one of which (p.R225W) located in the adult exon 6B. Clinical characterization of the family indicated that the cardiac disorder was present only in individuals with compound heterozygosity for the two mutations, with individuals carrying only one of the two Nav1.5 mutations being asymptomatic.^31^ This suggests an additive effect of the two mutations. To dissect the contribution of each mutation, we generated a hiPSC line from the patient and an isogenic corrected line carrying only the mutation in *SCN5A* exon 6B (hiPSC^corr/R225W^). However, when we recorded *I*_Na_ in hiPSC^corr/R225W^-CMs, current density was similar to that measured in WT hiPSC-CMs, whereas the compound mutant showed a reduction of *I*_Na_ density similar to that previously observed in hiPSC-CMs with only p.W156X mutation.^13^ The lack of effect of p.R225W contrasts with the strong reduction in current density shown in *Xenopus laevis* oocytes overexpressing the same p.R225W Nav1.5 mutation^31^; this could be partly due to differences between heterologous expression systems and human cells. Interestingly, the single mutation effect in patients was negligible, suggesting that there might be differences between *in vitro* systems and the patient phenotype. Analysis of *SCN5A* exon expression revealed that only a small fraction of exon 6B-containing transcripts was expressed in hiPSC-CMs, in line with previous reports in independent hiPSC lines.^29^ This explained the functional results and confirmed that the immature hiPSC-CM phenotype precludes analysis of mutations located in the adult *SCN5A* isoform.

Remarkably, while genetic excision of *Scn5a* exon 6B resulted in selective re-expression of foetal Nav1.5 in mice,^48^ our complementary approach of *SCN5A* exon 6A excision in hiPSCs did not lead to selective expression of the adult Nav1.5. Indeed, 6A-KO hiPSC-CMs expressed similar (low) amounts of *SCN5A* exon 6B compared with the original non-excised lines and presented negligible *I*_Na_. This demonstrates that the exon 6B expression levels in standard monolayer hiPSC-CM cultures are not sufficient to generate *I*_Na_ and that *I*_Na_ measured in non-excised hiPSC-CMs is mainly conducted by the foetal channel. The extremely low (or negligible) *I*_Na_ in 6A-KO hiPSC-CMs is likely a consequence of very few functional channels formed, because 6A excision interferes with the splicing machinery, as shown by the inclusion of intronic regions and exclusion of exon 5. This indicates that some regulatory sequences important for correct splicing might be present either within the intronic region removed by the excision, or in exon 6B itself, driving its own exclusion during splicing. Further studies with consecutive removal of different sequences within the 6A region are needed to elucidate the exact mechanisms underlying the regulation of *SCN5A* splicing. However, the switch between exon 6A and exon 6B is likely to need not only dedicated regulatory sequences, but also specific factors which may be lacking in immature CMs.

We and others developed different systems to improve the maturation of hiPSC-CMs using more physiological environments involving 3D engineering and/or co-culture with other (cardiac) cell types.^49,50^ We previously showed that hiPSC-CMs in our cardiac tri-cell-type MTs undergo maturation at the functional, structural, metabolic, and gene expression levels^30^; however, we did not examine whether it involved alternative splicing transition. Both ddPCR and RNA-seq revealed that in cardiac MTs there is a significant increase in the fraction of *SCN5A* exon 6B- vs. exon 6A-containing transcripts. A similar switch between *SCN5A* exon 6A and exon 6B was also previously promoted by culturing hiPSC-CMs in monolayer for extended periods.^29^ Of note, cardiac MT maturation is less time-consuming than prolonged monolayer culture (40 days vs. more than 60), and the number and ratio of cells in MTs remain unchanged during the 21 days of culture required for maturation.^30^ In our experience, long-term culture of hiPSC-CMs was accompanied by some cell loss, the proliferation of non-cardiomyocyte cells and the risk of detachment of the beating monolayer from the dish. Importantly, cardiac MTs showed higher expression of the exon 6B fraction (five- to six-fold, MT vs. 20 days 2D hiPSC-CMs) compared with long-term monolayer hiPSC-CM culture (two- to three-fold, 60 days vs. 20 days).^29^ Nevertheless, levels of exon 6B expression comparable to adult human heart were not reached, suggesting that splicing regulation may still differ between *in vitro* and *in vivo* conditions. It remains to be investigated whether the effects of exon 6B mutation are revealed in other 3D or monolayer maturation systems or media.

The possibility to derive all cardiac MT cell components from hiPSCs allowed us to include either mutant, corrected isogenic, or WT hiPSC-CMs while the other cell types derived from the same (WT) stock, avoiding possible confounding factors. Since hiPSC-CM maturation achieved in MTs is maintained after dissociation,^30^ we could analyse the electrical properties of single CMs. This revealed the contribution of p.R225W Nav1.5 mutation to the disease phenotype, uncovering the gene-dosage relationship and linking the presence of one or two mutated *SCN5A* alleles with a mild (∼45%) or severe (∼70%) *I*_Na_ reduction, respectively. Reduced *I*_Na_ density but no differences in the channel kinetics of activation or inactivation were measured in the p.R225W hiPSC-CMs compared with the corrected or WT hiPSC-CMs, much as has been observed for another mutation (p.I230T) in the same exon.^29^ A positive shift in the voltage dependence of *I*_Na_ activation was instead measured in the double mutant hiPSC-CMs, similar to the effects of the homozygous p.I230T Nav1.5 mutation.^29^ This shift can be attributed to the p.R225W mutation carried on the maternal—and only functional—allele, since the p.W156X mutation likely generates a non-functional protein, due to the stop codon in the paternal allele. In hiPSC^corr/R225W^, this change was instead masked by the corrected (and functional) allele. A shift in the voltage dependence of p.R225W Nav1.5 was also observed in transfected *X. laevis* oocytes^31^; however, this was more pronounced and involved both activation and inactivation, underscoring the fact that ion channel properties are influenced by species-specific cellular context (for example ancillary proteins/subunits).

The comparison between immature hiPSC-CMs and more mature CMs derived from MTs allowed us to investigate the mechanism underlying the switch between foetal and adult SCN5A isoforms. Mbnl1 was previously identified as necessary for exon 6B expression in mice, as the *Mbnl1*-knockout mice expressed only the foetal isoform of *SCN5A* in the heart.^7,10^ Here, we showed that *MBNL1* expression is low in 2D hiPSC-CM monotypic cultures, while its expression increases significantly when CMs are included in MTs. Interestingly, only *MBNL1* was specifically up-regulated in the CM population, and not *MBNL2*, another member of the same family. *MBNL1* is also characterized by different developmentally regulated splicing isoforms and it is known to regulate its own splicing.^44,51^ In particular, exon 5 is included in the embryonic isoform and is gradually excluded postnatally.^51,52^ In MTs, we observed reduced expression of *MBNL1* exon 5 compared with monolayer hiPSC-CMs, suggesting a functional change in MBNL1. Overall this indicates that *MBNL1* is up-regulated and alternatively spliced in mature hiPSC-CMs and it might be specifically required to induce the switch in *SCN5A* isoform. Indeed, transient overexpression of *MBNL1* increased *SCN5A* exon 6B fraction in hiPSC-CMs and lack of *MBNL1* prevented splicing isoform switch in MTs. This confirms a key role for this factor in regulating *SCN5A* splicing also in human cells. Since we performed transient expression of *MBNL1* in hiPSC-CMs, it remains to be elucidated whether the sole expression of the adult *MBNL1* is sufficient to promote functional maturation of Nav1.5. Moreover, further studies are needed to understand how *MBNL1* overexpression and knockout influence the expression of other target genes beside *SCN5A*. Indeed, Mbnl1 was shown to regulate the post-natal isoform switch of many other genes during mouse heart development^7–9^ and to repress human stem cell pluripotency while promoting differentiation.^53,54^ The expression of adult *MBNL1* in hiPSC-CMs within the MT opens the possibility to study other developmentally/postnatally regulated genes using hiPSC-derived cells. The alternative splicing of the region of the voltage-sensing S4 segment is conserved also in voltage-gated sodium channels expressed in the brain, generating different isoforms underlying functional differences between foetal and adult tissues.^55^ It is tempting to speculate that promoting hiPSC maturation in 3D organ-specific tissues might facilitate the study of adult disease phenotypes *in vitro* in other organs.

In conclusion, our data demonstrate that a post-natal maturation of hiPSC-CMs is required to express the adult *SCN5A* isoform through MBNL1 regulation and thus reveal mutation contributions, allowing dissection of ionic current changes that cause adult arrhythmic disease phenotypes in humans.

## Supplementary material

Supplementary material is available at *Cardiovascular Research* online.

## Authors’ contributions

M.B., C.L.M., and G.C.: conceptualization; G.C., G.K, A.O.V, D.O., and H.M.: formal analysis; C.L.M., M.B., and G.C.: funding acquisition; G.C., G.K., D.W.-v.O., L.Y., and A.O.V.: investigation; D.W.-v.O., M.B., and G.C.: methodology; M.B.: project administration; A.A.M.W., C.R.B., and C.C.V.: resources; M.B., A.O.V., R.P.D., and V.V.O.: supervision; and G.C., M.B., and C.L.M.: writing—original draft.

## Supplementary Material

cvac059_Supplementary_Data

## Data Availability

Data are incorporated into the article and its Supplementary material online. RNA-seq raw data are deposited in GEO under the accession number GSE180290. All raw data are available from the corresponding author upon request.
