# Supplementary material for: Maturation of hiPSC-derived cardiomyocytes promotes adult alternative splicing of SCN5A and reveals changes in sodium current associated with cardiac arrhythmia
Source: Cardiovasc Res. 2022 Apr 8;119(1):167–82. doi: 10.1093/cvr/cvac059 (PMC10022870; doi:10.1093/cvr/cvac059)
Supplement: cvac059_Supplementary_Data [file cvac059_supplementary_data.zip › Supplement_R1.pdf]

Supplementary Material for

Maturation of hiPSC-derived cardiomyocytes promotes adult alternative splicing of SCN5A and reveals changes in sodium current associated with cardiac arrhythmia

Campostrini et al.

Corresponding author: Milena Bellin
Email: m.bellin@lumc.nl or milena.bellin@unipd.it

This PDF file includes:

Supplementary figures (S1-S8) and tables (S1-S3)
Supplementary methods

Supplementary Figures and Figure Legends

Figure S1

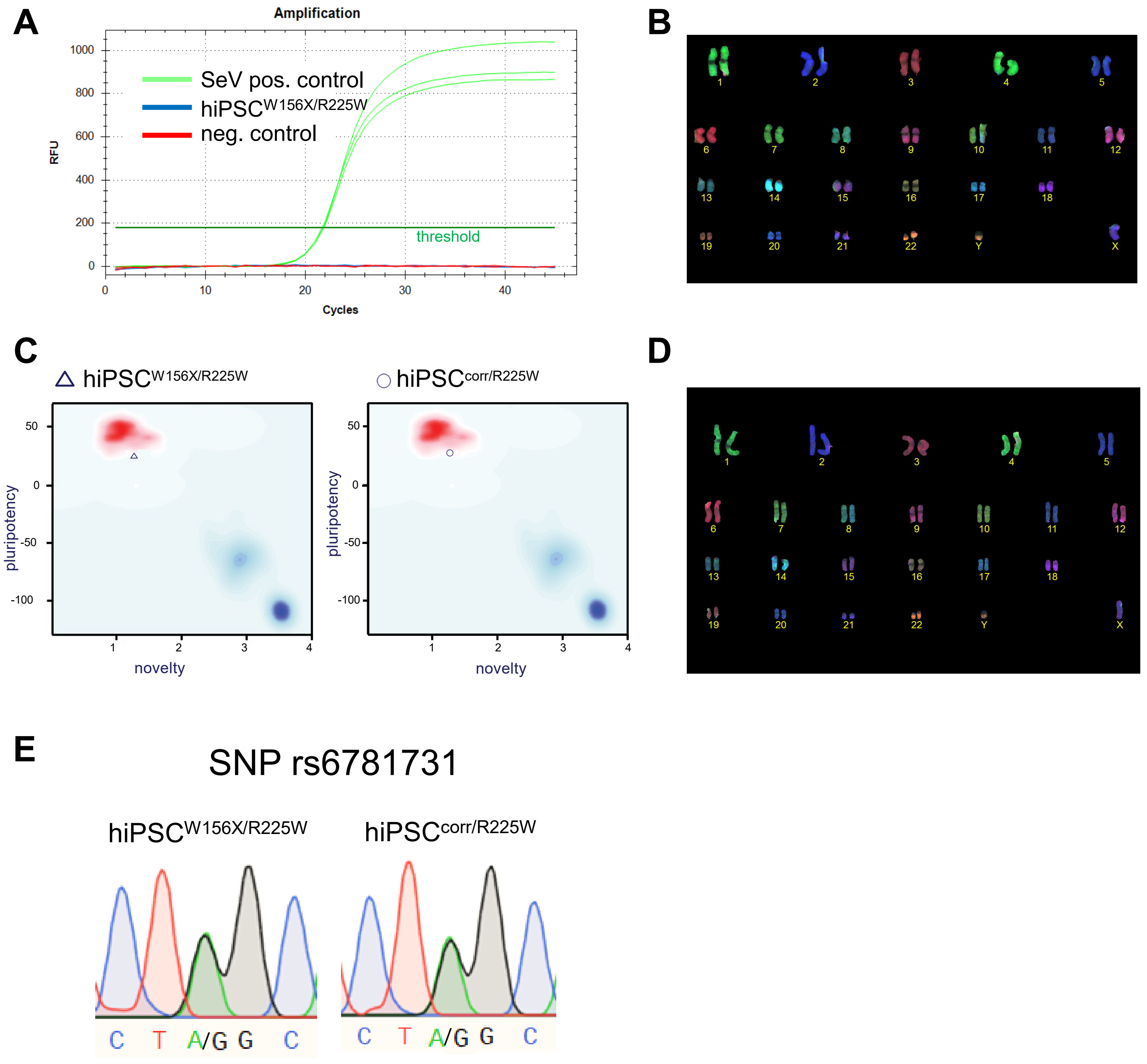

Genetic characterization of hiPSC$^{W156X/R225W}$ and hiPSC$^{corr/R225W}$. A. qPCR analysis for Sendai virus (SeV). SeV positive control, hiPSC$^{W156X/R225W}$, and negative control are shown. **B.** Karyotype analysis in hiPSC$^{W156X/R225W}$ line shows no abnormalities. **C.** Results of PluriTest showing that both hiPSC lines cluster together with human pluripotent stem cells (red cloud). **D.** Karyotype analysis in hiPSC$^{corr/R225W}$ line showing no abnormalities. **E.** Representative Sanger sequence chromatograms of the region downstream *SCN5A* exon 4, showing the heterozygous rs6781731 SNP present in both hiPSC$^{W156X/R225W}$ and hiPSC$^{corr/R225W}$ lines.

Figure S2

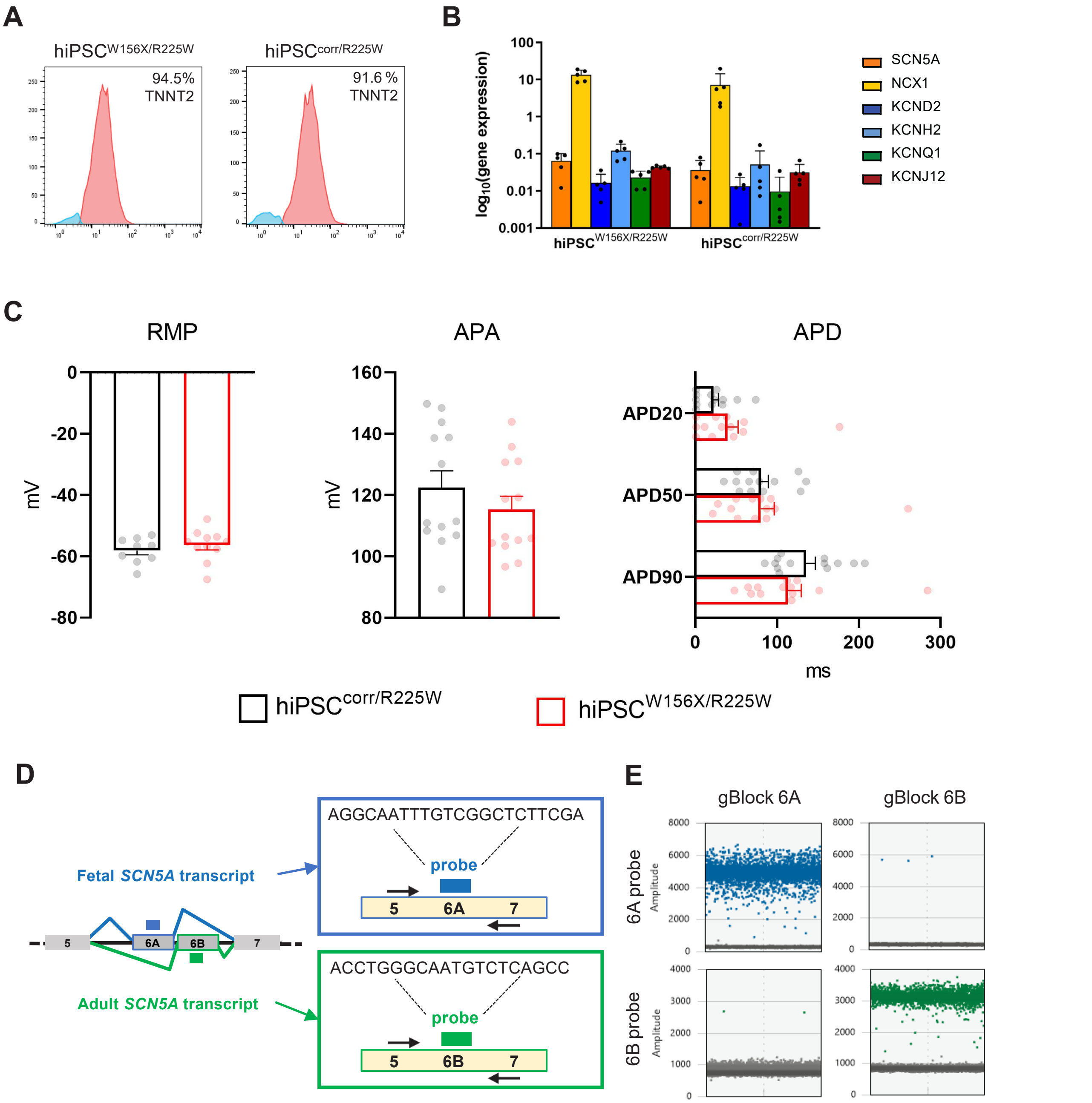

Gene expression and AP characteristics of hiPSC-CMs from 2D cultures. A. Representative FACS analysis on CMs from hiPSC$^{W156X/R225W}$ and hiPSC$^{corr/R225W}$ lines showing that >90% of the cell population is TNNT2^{+}. **B.** Gene expression analysis (qPCR) of several cardiac ion channels, as indicated, in hiPSC$^{W156X/R225W}$- and hiPSC$^{corr/R225W}$-CMs. n=5, $p>0.05$ with Student's t-test. Dots: single values. **C.** Action potential parameters measured in single CMs differentiated from hiPSC$^{corr/R225W}$ (black) and hiPSC$^{W156X/R225W}$ (red) lines. Data are presented as mean±SEM. Dots: single values. RMP: spontaneous resting membrane potential; APA: action potential amplitude; APD: action potential duration. **D.** Schematic showing the design of the ddPCR assay: specific probes for exon 6A (blue) and exon 6B (green) conjugated to two different fluorophores were used to identify fetal and adult transcripts, respectively. Reaction was performed using a single pair of primers. Probe sequences and the position of primers are indicated. **E.** Specificity of the two probes was evaluated by adding an equal amount of both in a reaction using as template synthetic (gBlock) exon 6A or 6B sequences.

Figure S3

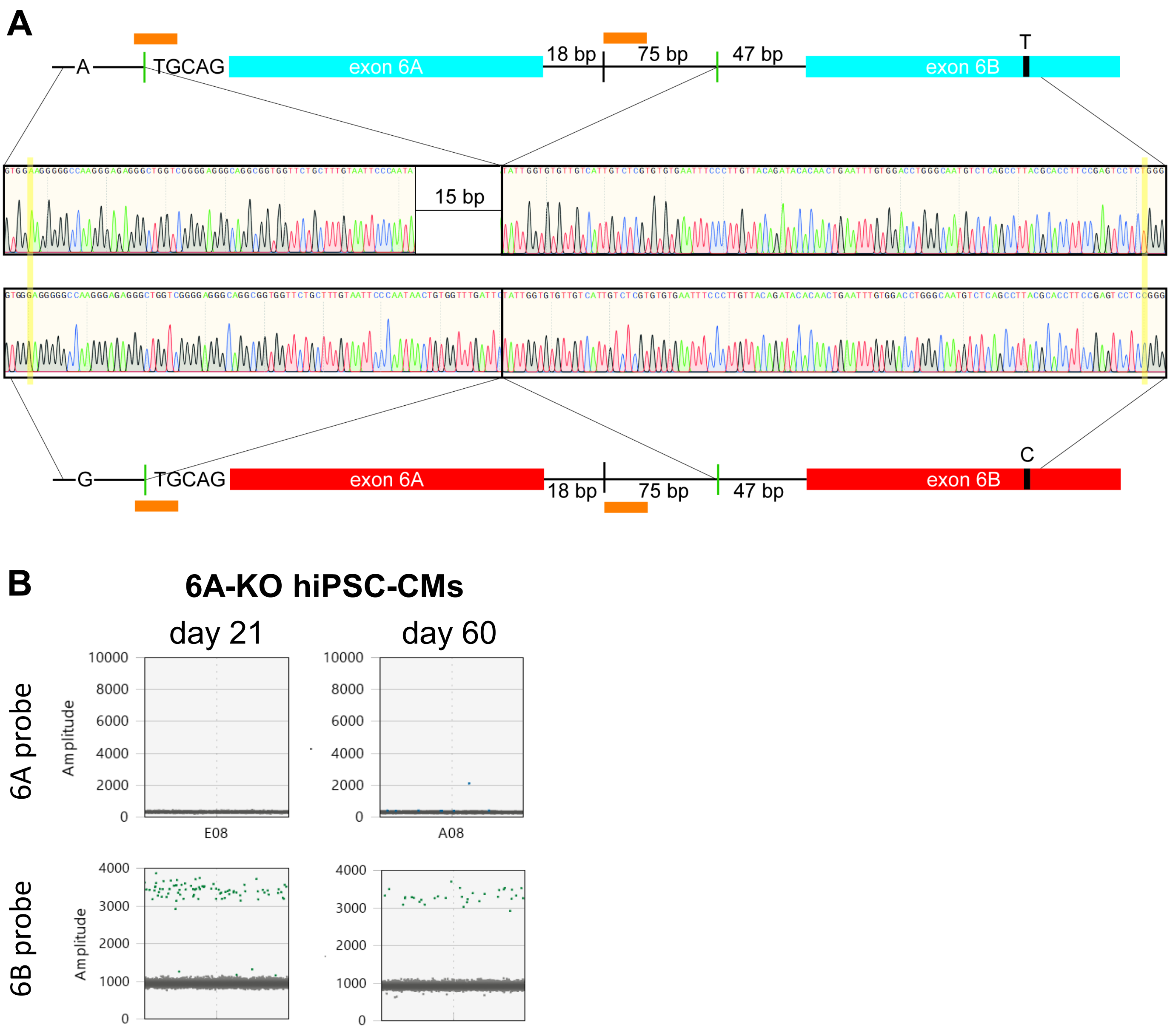

***SCN5A* exon 6A excision in hiPSCs$^{W156X/R225W}$. A.** Schematic displaying results of the CRISPR/Cas9 excision of *SCN5A* exon 6A in hiPSC$^{W156X/R225W}$ line. The maternal (blue) and paternal (red) *SCN5A* alleles are shown, along with the corresponding chromatograms of Sanger sequencing after excision. Orange boxes indicate sgRNAs used for the excision. In the maternal allele, the excision upstream of exon 6A was 15 bp larger than in the paternal allele. The actual cutting site downstream exon 6A was in both alleles 75 bp downstream of the PAM sequence of the sgRNA located in intron 6A-6B. The rs6781731 SNP and the mutation in exon 6B are highlighted in yellow in the chromatogram. **B.** Representative ddPCR dot plots showing expression of exon 6B but no expression of exon 6A in 6A-KO hiPSC-CMs both at 21 and 60 days of culture.

Figure S4

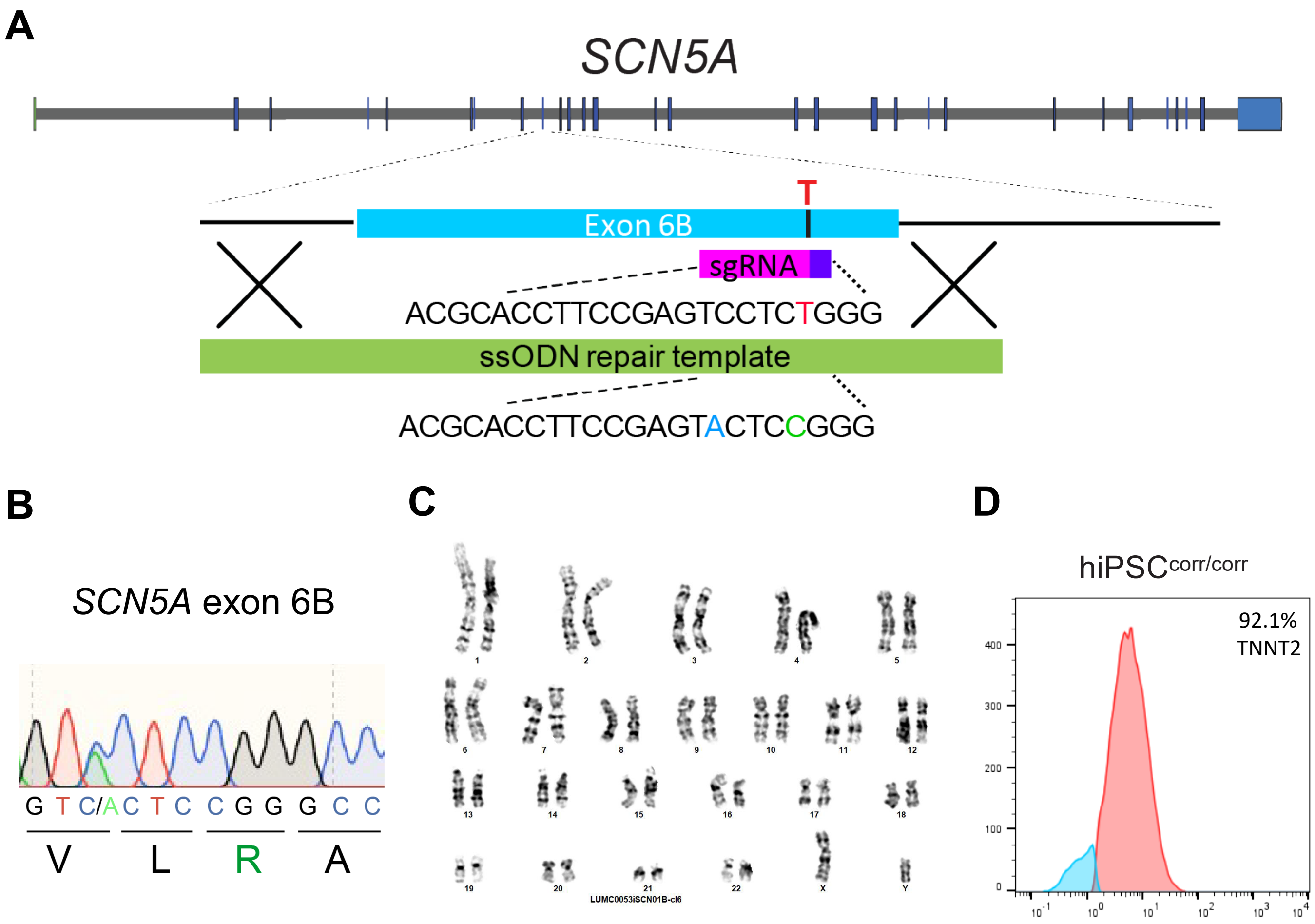

Correction of p.R225W mutation in *SCN5A* exon 6B and characterization of hiPSCs$^{corr/corr}$. A. Schematic showing the strategy used to correct the c.673C>T (p.R225W) mutation in SCN5A exon 6B with CRISPR/Cas9 in the maternal allele of hiPSC$^{corr/R225W}$. The mutant timine base is shown in red; in pink the sgRNA guiding the Cas9 to the mutation; in green the ssODN used as donor template for homology directed DNA repair. Underneath part of the ssODN sequence, showing in green the wild-type cytosine base and in light blue the silent mutation. **B.** Sanger sequencing chromatograms showing part of SCN5A exon 6B after correction: the patient mutation is corrected and a silent mutations is introduced in one allele. **C.** Karyotype analysis in hiPSC$^{corr/corr}$ line showing no abnormalities. **D.** Representative FACS analysis on CMs from hiPSC$^{corr/corr}$ showing that >90% of the cell population is TNNT2^{+}.

Figure S5

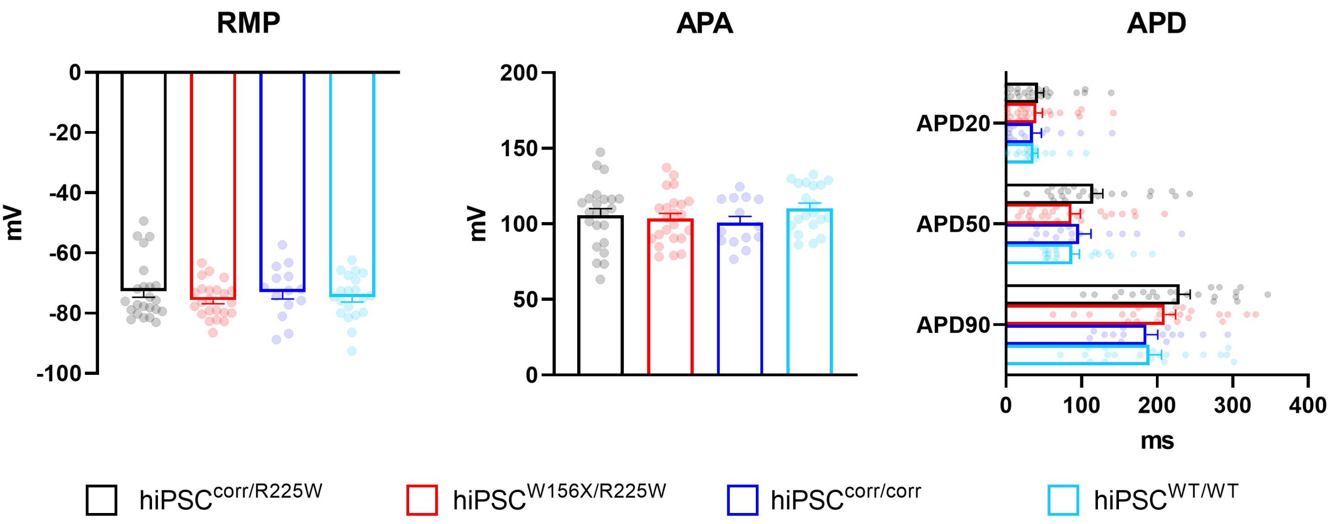

AP parameters analysed in MTs. Parameters of AP stimulated at 1Hz measured before applying the dynamic clamp in single CMs dissociated from MTs and differentiated from hiPSC$^{corr/R225W}$ (black), hiPSC$^{W156X/R225W}$ (red), hiPSC$^{corr/corr}$ (blue), and hiPSC$^{WT/WT}$ (light blue), lines. Dots: single values. RMP: resting membrane potential; APA: action potential amplitude; APD: action potential duration.

Figure S6

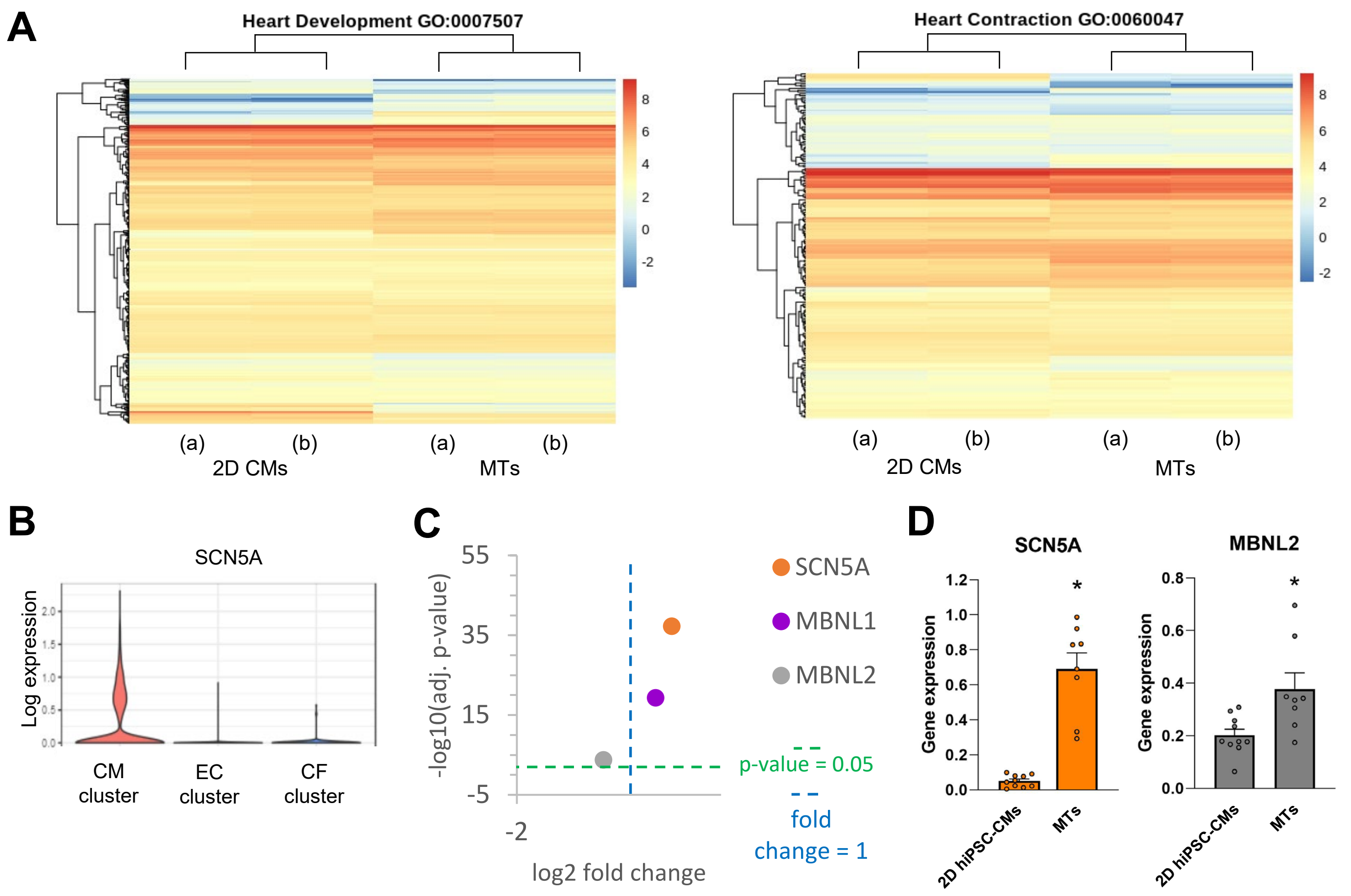

Gene expression in hiPSC-CMs and MTs evaluated by RNA-seq. A. Heat map showing log2 expression of genes belonging to GO terms of "heart development" and "heart contraction" in 2D CMs and MTs from (a) hiPSC$^{corr/R225W}$ and (b) hiPSC$^{W156X/R225W}$. **B.** ScRNA-seq analysis in MTs from hiPSC$^{WT/WT}$ (data from Giacomelli et al. 2020) showing *SCN5A* expression specifically in CM cluster. **C.** Plot of sorted log2 fold-change vs. adjusted p-values for gene expression of *SCN5A*, *MBNL1*, and *MBNL2* in hiPSC-CMs from MTs compared to 2D cultured hiPSC-CMs based on their scRNA-seq data reported in Giacomelli et al. 2020. **D.** Gene expression analysis by qPCR for *SCN5A* and *MBNL2* in hiPSC-CMs from 2D cultures (n=10) and MTs (n=8). *$p<0.05$, Student's t-test. Dots: single values.

Figure S7

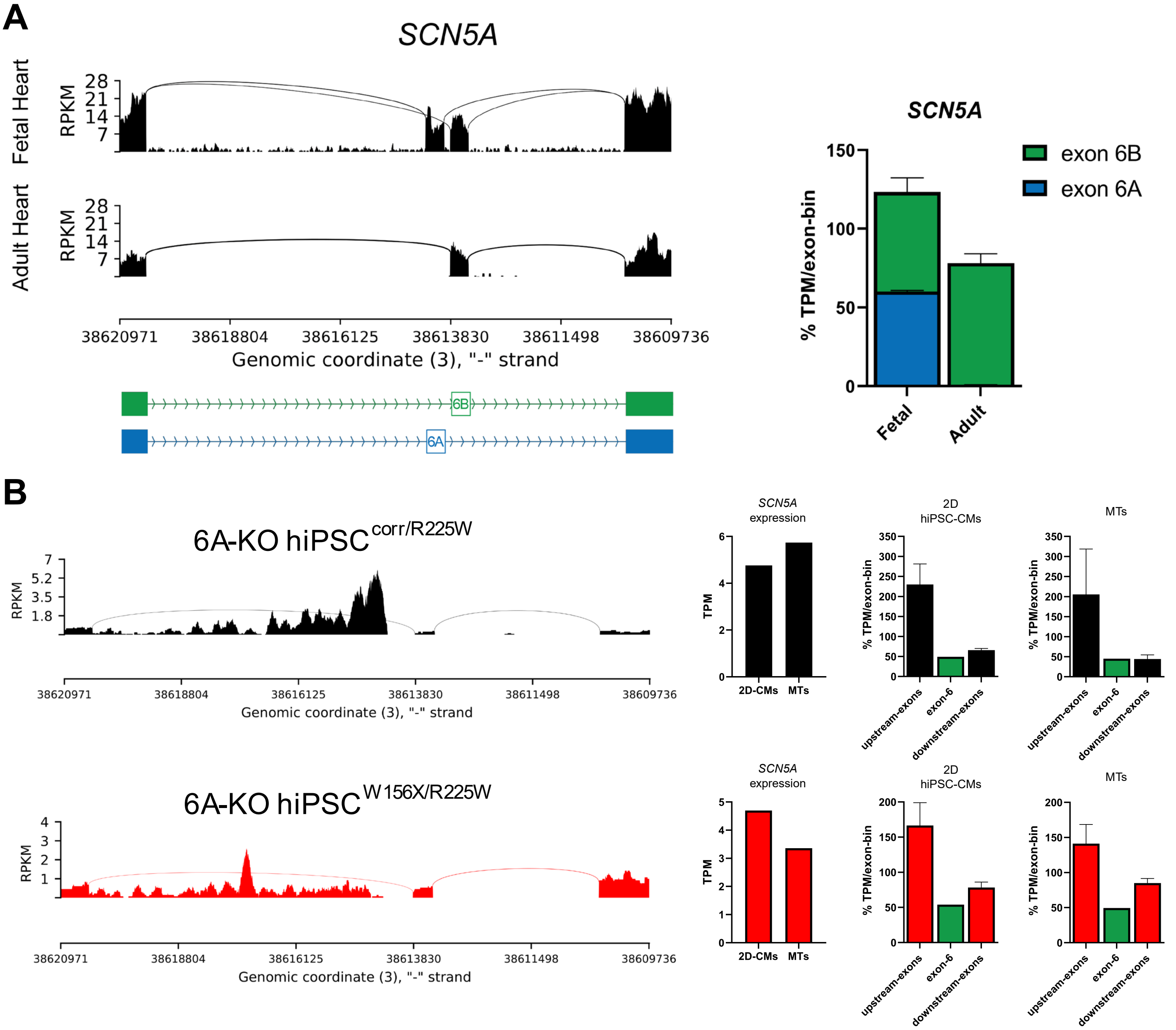

RNA-seq analysis of SCN5A alternative splicing of exon 6 in fetal and adult heart and in 6A-KO lines. A. Sashimi plots (left) and bar graphs (right) showing expression of exon 6A (blue) and 6B (green) analysed by RNA-seq in fetal and adult hearts (data from Kuppusamy et al. 2015, GEO: GSE62913). **B.** RNA-seq data from 6A-KO MTs and 2D hiPSC-CMs. Left, sashimi plots showing transcription in the intronic region between exon 5 and exon 6B. Right, bar graphs showing no increase of SCN5A expression in MTs compared to 2D hiPSC-CMs in both lines and a reduction in transcription of exons downstream to exon 6 in both 2D hiPSC-CMs and MTs.

Figure S8

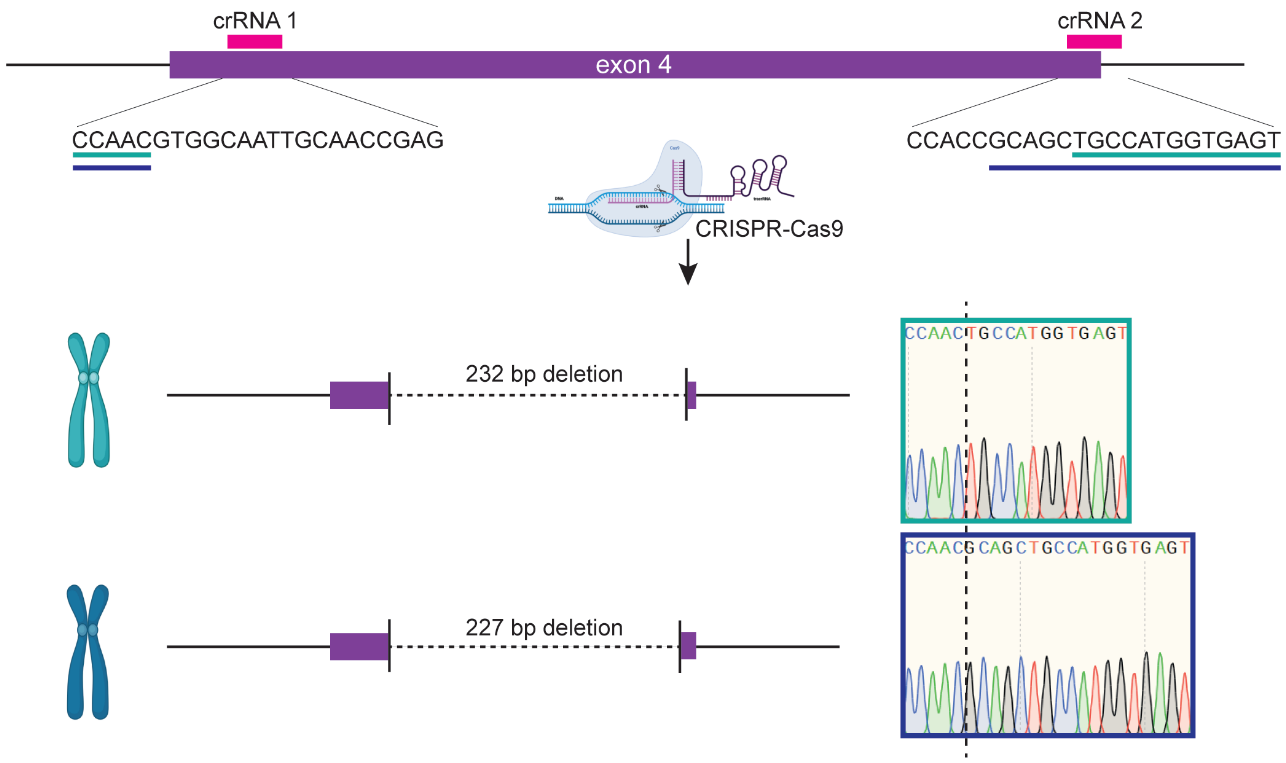

Genetic knockout of MBNL1. Schematics of the deletion of a large part of exon 4 of MBNL1 in hiPSC$^{corr/R225W}$, resulting in a frameshift. Two crRNA (top, pink) have been used to promote excision by CRISPR-Cas9 and their sequence is indicated. Both alleles (light and dark blue) were excised, as indicated, with a different deletion extent. Sanger sequencing from the topocloning corresponding to each allele is shown on the right. Vertical dotted line indicates the cutting site.

Supplementary Tables

Table S1. Sequences of sgRNA and ssODN used for CRISPR/Cas9 gene editing

Name	Sequence
sgRNA exon 4	CACTCGACATACTTGGTTCA
sgRNA#1 exon 6A	ACTTGTAGCTGAGATCTGAG
sgRNA#2 exon 6A	CAGAATCAAACCACAGTTAT
Alt-R crRNA exon 6B	ACGCACCTTCCGAGTCCTCT
Alt-R crRNA MBNL1 exon 4 (1)	CTCGGTTGCAATTGCCACGTTGG
Alt-R crRNA MBNL1 exon 4 (2)	ACTCACCATGGCAGCTGCGGTGG
ssODN exon 4	CATGTGCACCATCCTCACCAACTGCGTGTTCATGGCCCAGCACGACCCTCCACCTTGGACCAAATACGTGGAGTGAGTATCTTCAGGGCCTCTTCTCCACGTGGCCCCCTCCCTTCCTTTCCATTC
ssODN exon 6B	AATTTCCCTTGTTACAGATACACAACTGAATTTGTGGACCTGGGCAATGTCTCAGCCTTACGCACCTTCCGAGTCCTCCGGGCCCTGAAAACTATATCAGTCATTTCAGGTGAAAATCAG

Table S2. Primers used for RT-PCR, ddPCr, and q-RT-PCR

Name	Forward	Reverse	Use
exon 4 region	CTGCTCAGCTTTCCTTGACC	TCCTCCCTAGAAGGCACAAC	RT-PCR
exon 6A/exon 6B region	GGAGGGTTGAAATCCAGAAG	ATTCTGGTGACAGGCACATT	RT-PCR
ddPCR exon 6	ACTGGCTGGACTTTAGTGTG	CCCACGATGGTCTTCAGC	ddPCR
RPL37A	GTGGTTCCTGCATGAAGACAGTG	TTCTGATGGCGGACTTTACCG	q-RT-PCR
SCN5A	GAGCTCTGTCACGATTTGAGG	GAAGATGAGGCAGACGAGGA	q-RT-PCR
NCX1	ACATCTGGAGCTCGAGGAAA	CTGGAATTCGAGCTCTCCAC	q-RT-PCR
KCND2	TCAGATGTGTGGAGAGAACACC	CCTTCTGGTGTGGTTACTGGA	q-RT-PCR
KCNH2	CACCGCCCTGTACTTCATCT	AGGCCTTGCATACAGGTTCA	q-RT-PCR
KCNQ1	TCCTGGTCTGCCTCATCTTC	AAGAACACCACCAGCACGAT	q-RT-PCR
KCNJ12	TGGATCCTTTCCAGTTGGTG	CGGCTCCTCTTGAGTTCTATCTT	q-RT-PCR
MBNL1	GGACGAGTAATCGCCTGCTTT	TCTGCTGAATCAAGTTATTGCGT	q-RT-PCR
MBNL2	TCAAAGAGGAACATGCTCACG	AACGGCCCTTTAGGGAATCAA	q-RT-PCR
exon 6B region	GGAGGGTTGAAATCCAGAAG	ATTCTGGTGACAGGCACATT	RT-PCR
MBNL1 exon 4	TGCTGAAGTTACTTGTTGCCT	TGTCTGGGATACCTGAACAAAC	RT-PCR

Table S3. Probes used for ddPCR

Name	ddPCR probe sequence
exon 6B	ACCTGGGCAATGTCTCAGCC
exon 6A	AGGCAATTTGTCGGCTCTTCGA

Supplementary methods

hiPSC culture and in vitro cardiac differentiation

Patient hiPSCs were initially cultured on mouse embryonic feeder (MEF) cells plated on gelatin-coated plates in hiPSC growth medium containing DMEM:F12 1:1, 20% knockout serum, 1X MEM non-essential amino acids, 1X 2-mercapto-ethanol (all from ThermoFisher Scientific), 10 ng/mL human FGF-2 (Miltenyi Biotec), and then adapted to feeder-free culture. All hiPSC lines were cultured on recombinant human vitronectin-coated plates in Essential 8 medium (ThermoFisher Scientific) following the manufacturer's protocol and passaged twice weekly as previously described[1, 2]. hiPSC lines were routinely tested for mycoplasma. Pluripotency was confirmed by immunofluorescence and by using the Pluritest algorithm (GenomeScan BV) on total RNA isolated from the hiPSCs. Karyotyping of hiPSCs was performed by G-banding by the Laboratory for Diagnostic Genome Analysis (Leiden University Medical Center). Cardiac differentiation was induced in monolayer culture, as previously described[2, 3]. Briefly, 24 h before starting differentiation, 2.5×10^4 cells per cm^2 were plated on Matrigel (Corning)-coated plates. At day 0 of differentiation, a mixture of cytokines (20 ng/mL BMP4, R&D; 20 ng/mL Activin A, Miltenyi Biotec) and the GSK3 inhibitor CHIR99021 (1.5 μmol/L, Axon Medchem) in LI-BPEL medium[4, 5] was added to the cells to induce mesoderm formation. After 2-3 days, the cytokines were removed and a Wnt inhibitor (5 μmol/L, XAV939, TOCRIS) in LI-BPEL medium was added for 3 days. LI-BPEL medium was further refreshed every 3–4 days.

Cardiac endothelial cells (ECs) and cardiac fibroblasts (CFs) were obtained from the $hiPSC^{WT/WT}$ line and cryopreserved, as previously described[2]. Briefly, for ECs, VEGF (50 ng/ml, R&D Systems) and XAV939 (5 μmol/L) were added after mesoderm induction to LI-BPEL medium for 3 days and ECs subsequently isolated using Human cord blood CD34 Positive selection kit II (StemCell Technologies). ECs were then plated onto fibronectin-coated plates in LI-BPEL medium supplemented with VEGF (50 ng/ml). CFs were derived from hiPSC-derived epicardial cells (EPI).

EPI were differentiated through mesoderm induction by culturing hiPSCs in LI-BPEL supplemented with XAV939 (5 µmol/L), BMP4 (30 ng/mL) and retinoic acid (1 µmol/L) for 3 days, then for another 3 days in the same medium without XAV939; the cells were subsequently plated on fibronectin-coated plates in LI-BPEL supplemented with TGFβ inhibitor SB431542 (10 µmol/L). CF specification was induced by plating EPI on vitronectin-coated plates in LI-BPEL medium supplemented with FGF-2 (10 ng/mL). After 6 days, CFs were expanded in Fibroblast Growth Medium 3 (PromoCell).

DNA sequencing

Genomic DNA was isolated from hiPSCs using Quick Extract (Lucigen Biosearch Technologies) or Gentra PureGene Cell Kit (Qiagen). The genomic regions surrounding mutations W156X and R225W were amplified by PCR using SilverStar DNA polymerase (Eurogentec) or Platinum Taq High Fidelity (ThermoFisher Scientific) and specific primer sets (Table S1). PCR products were purified using the QIAquick PCR Purification kit (Qiagen) and sequenced with Sanger sequencing.

CRISPR/Cas9 editing of hiPSCs and clonal isolation

hiPSC$^{W156X/R225W}$ maintained on MEFs or 6A-KO hiPSC$^{W156X/R225W}$ in feeder-free culture were used for transfection using 1 µL/cm^2 Lipofectamine 2000 diluted in Opti-MEM medium (ThermoFisher Scientific) along with the plasmid DNA (0.4 µg/cm^2). For the correction of c.468G>A mutation in exon 4 (p.W156X), a single-stranded DNA oligonucleotide (ssODN) repair template carrying the wild-type guanine base and four silent mutations (Table S1) (IDT technologies, 10 pmol) was used as the donor DNA template for homology directed repair. 24 h later, transfected cells were selected by adding puromycin (0.5 µg/ml) for 36 h. For the correction of c.673C>T mutation in exon 6B (p.R225W), hiPSC$^{corr/R225W}$ in feeder-free condition were electroporated with Alt-R® S.p. HiFi Cas9 Nuclease V3 (IDT technologies) combined with the complex of Alt-R-crRNA/Alt-R-tracrRNA and an ssODN repair template (IDT technologies) carrying the wild-type timine base and a silent mutation generating a Sca-I cutting site (Table S1 and Fig S4A). Clones were isolated by

limiting dilution in 96-well plates (0.7 cell/well). After 1-2 weeks, colonies were dissociated and expanded.

Microtissue formation and culture

Three days before MT formation, cryopreserved hiPSC-ECs and -CFs were thawed in their respective medium described above. hiPSC-ECs were plated on fibronectin-coated plates and hiPSC-CFs on uncoated plates. hiPSC-CMs were either thawed 5 days before MT formation and plated on Matrigel-coated plates or used fresh from differentiated cells at day 21. On the day of MT formation, cells were dissociated using either TrypLE Select 1X (ECs and CFs) or Multi Tissue Dissociation Kit 3 (Miltenyi Biotec) following the manufacturer's instructions. Cells were resuspended in LI-BPEL medium supplemented with VEGF (50 ng/ml) and FGF-2 (5 ng/ml), counted and combined in the defined ratios to obtain 100 cells/µL. 50 µL/well of the cell suspension were plated in V-bottomed 96-well plates (Greiner Bio-One) and centrifuged for 10 min at 1100 rpm, to promote aggregation. MTs were kept in a humidified incubator at 37°C, 5% CO_2 for 21 days and medium was refreshed every 3-4 days, until analysis.

Flow cytometry

The efficiency of cardiomyocyte differentiation at day 19-21 was determined by flow cytometry using cardiac troponin T (TNNT2) staining on fixed cells. Briefly, cardiomyocytes were dissociated with Miltenyi Biotec Multi Tissue dissociation kit 3, fixed and permeabilized with Fix and perm reagent A and B (ThermoFisher Scientific), followed by TNNT2 staining with Cardiac Troponin T-VioBlue (1:10 or 1:25, Miltenyi Biotec). Samples were measured using a MACSQuant VYB flow cytometer (Miltenyi Biotec), and data analysed using using a MACSQuant VYB flow cytometer (Miltenyi Biotec), and data analysed using FlowJo (Tree star).

Immunofluorescence analysis

hiPSCs and hiPSC-CMs were fixed in 4% paraformaldehyde, permeabilized with phosphate buffer saline (PBS)/0.1% Triton X-100 (Sigma-Aldrich), and blocked with 10% Fetal Calf Serum (FCS, ThermoFisher Scientific). Samples were incubated overnight at 4°C with primary antibodies as follows. For hiPSCs, cells were stained with antibodies for NANOG (rabbit polyclonal, Peprotech), SSEA4 (mouse monoclonal, Biolegend) and POU5F1 (mouse monoclonal, Santa Cruz), and detected with Alexa-Fluor 488-, Alexa 568- or Cy3-conjugated antibodies respectively. For hiPSC-CMs, cells were stained with antibodies for TNNT2 (rabbit polyclonal, Abcam #ab45932, dilution 1:1500) and ACTN2 (mouse monoclonal, Sigma-Aldrich #A7811, dilution 1:1000). Primary antibodies were detected with either Alexa-Fluor 488 or Cy3-conjugated antibodies. Nuclei were visualized with DAPI (Invitrogen). Images were acquired using a Leica DMI6000-AF6000 fluorescence microscope (for hiPSCs) or Leica SP8 WLL confocal laser-scanning microscope using a 63x magnification objective and Z stack acquisition (for hiPSC-CMs).

Patch clamp

APs recorded by perforated patch clamp were low-pass filtered with a cut-off of 2 kHz and digitized at 10 kHz. hiPSC-CMs were kept at 37°C and perfused with Tyrode solution containing (in mmol/L): 140 NaCl, 5.4 KCl, 1.8 $CaCl_2$, 1 $MgCl_2$, 5.5 D-Glucose, 5 HEPES-NaOH, pH 7.4 (set with NaOH). Patch pipettes (resistance 2–3 MΩ; Harvard Apparatus, UK) were filled with solution containing (in mmol/L): 125 K-gluconate, 20 KCl, 10 NaCl, 10 HEPES, 0.52 amphotericin-B, pH 7.2 (set with KOH). APs were elicited at 1 Hz with square 2 ms, ~1.2× threshold current pulses through the patch pipette. Data from 10 APs were averaged for the analysis of AP parameters. Voltages were corrected for the calculated liquid junction potential of 15 mV.

I_{Na} signals were low-pass filtered with a cut-off of 5 kHz and digitized at 28.57 kHz. Cardiomyocytes were kept at room temperature (20°C) and perfused with a reduced Na^+ concentration solution containing (in mmol/L): 20 NaCl, 120 CsCl, 1.8 $CaCl_2$, 1.2 $MgCl_2$, 11 D-Glucose, 5 HEPES, nifedipine 0.01, pH 7.4 (set with CsOH). Patch clamp pipettes had a resistance

of 1.8–2.5 MΩ and were filled with (in mM): 3 NaCl, 133 CsCl, 2 $MgCl_2$, 2 Na_2ATP, 2 TEACl, 10 EGTA, 5 HEPES, pH 7.2 (set with CSOH). Series resistance was compensated by ≥ 75%. Calculated liquid junction potential was negligible and correction was not applied. I_{Na} was measured by 50 ms depolarizing voltage steps between -80 and +40 mV (with 5 mV increment) from holding potential of -100 mV and at a cycle length of 4 s. Current densities were calculated dividing current intensity by cell capacitance. Activation curves were obtained from normalized conductance at each voltage step and inactivation curves were obtained from normalized tail currents at -20 mV preceded by a 500 ms conditional step ranging from -120 to -25 mV. Curves were fitted with a Boltzmann equation ($I/I_{max}=A/(1.0+\exp((V_{1/2}-V)/k))$), where $V_{1/2}$ is the voltage at half-maximal (in)activation, and k is the slope factor.

ddPCR analysis of SCN5A exon 6A and 6B expression

Primers were designed to amplify both splicing variants, whereas probes were specific to either 6A or 6B exon and were carrying either FAM (6A) or HEX-fluorophore (6B) for detection. Probe specificity was determined using 6A- or 6B- synthetic sequences (gBlock, IDT) as DNA template for ddPCR (Fig S2E). Fraction of 6A and 6B expression was calculated based on copies/µL, and the sum of 6A and 6B expression was considered as the total. Expression levels were normalized to the housekeeping TATA-box binding protein gene (*TBP*), to compare gene expression between 2D cultured hiPSC-CMs and MTs. Human adult and fetal (25-40 weeks) Heart- total RNA (Clontech) were used as a reference.

MBNL1 mRNA in vitro transcription and transfection

MBNL1 sequence was ordered as a gBlock (IDT), containing the T7 promoter, target sequence, *T2A* self-cleaving peptide sequence and eGFP and cloned into the pMiniT 2.0 vector using the NEB PCR Cloning Kit (New England Biolabs) according to the manufacturer's instructions. The target gBlock sequence was PCR amplified using PrimeSTAR Max DNA polymerase (Takara) using the primers provided by the NEB PCR Cloning Kit (New England Biolabs). Following PCR

purification, the PCR product was digested with XhoI restriction enzyme giving a 5’ overhang at the 3’ end of template. The digested product was run for 2 h at 4°C on a 1% low-melt agarose TBE gel (Promega), gel extracted using the Wizard SV Gel and PCR Clean-Up System (Promega) and used as a template for in vitro transcription.

For transfection, 120 ng of the mRNA per 40,000 cells was mixed with Opti-MEM (Gibco) and 0.5 µl Lipofectamine Stem Transfection Reagent in a total volume of 40 µl and incubated for 10 min at RT before being added drop-wise to the cells. The cells were refreshed with mBEL medium 18-20 h after transfection. Samples were collected 3 days after transfection and analysed for gene expression and transfection efficiency. For assessing transfection efficiency, cells were dissociated using 5X TrypLE Select (ThermoFisher Scientific) for 10 min at 37°C, resuspended in FACS wash buffer ($PBS^{-/-}$ supplemented with 2% FBS and 2 mM EDTA) and analysed with flow cytometry.

References

1. Giacomelli E, Meraviglia V, Campostrini G, Cochrane A, Cao X, Van Helden RWJ, Krotenberg Garcia A, Mircea M, Kostidis S, Davis RP, Van Meer BJ, Jost CR, Koster AJ, Mei H, Míguez DG, Mulder AA, Ledesma-Terrón M, Pompilio G, Sala L, Salvatori DCF, Slieker RC, Sommariva E, De Vries AAF, Giera M, Semrau S, Tertoolen LGJ, Orlova VV, Bellin M, Mummery CL. Human-iPSC-Derived Cardiac Stromal Cells Enhance Maturation in 3D Cardiac Microtissues and Reveal Non-cardiomyocyte Contributions to Heart Disease. *Cell Stem Cell* 2020.
2. Campostrini G, Meraviglia V, Giacomelli E, van Helden RWJ, Yiangou L, Davis RP, Bellin M, Orlova VV, Mummery CL. Generation, functional analysis and applications of isogenic three-dimensional self-aggregating cardiac microtissues from human pluripotent stem cells. *Nat Protoc* 2021;**16**:2213-2256.
3. van den Berg CW, Elliott DA, Braam SR, Mummery CL, Davis RP. Differentiation of Human Pluripotent Stem Cells to Cardiomyocytes Under Defined Conditions. *Methods Mol Biol* 2016;**1353**:163-180.
4. Elliott DA, Braam SR, Koutsis K, Ng ES, Jenny R, Lagerqvist EL, Biben C, Hatzistavrou T, Hirst CE, Yu QC, Skelton RJP, Ward-Van Oostwaard D, Lim SM, Khammy O, Li X, Hawes SM, Davis RP, Goulburn AL, Passier R, Prall OWJ, Haynes JM, Pouton CW, Kaye DM, Mummery CL, Elefanty AG, Stanley EG. NKX2-5eGFP/w hESCs for isolation of human cardiac progenitors and cardiomyocytes. *Nature Methods* 2011;**8**:1037-1040.
5. Sala L, Ward-van Oostwaard D, Tertoolen LGJ, Mummery CL, Bellin M. Electrophysiological Analysis of human Pluripotent Stem Cell-derived Cardiomyocytes (hPSC-CMs) Using Multi-electrode Arrays (MEAs). *J Vis Exp* 2017.
